# Membrane‐Active Peptide Protects Against Inflammation by Targeting NLRP3 Activation at the Trans‐Golgi Network

**DOI:** 10.1002/advs.76587

**Published:** 2026-07-21

**Authors:** Jonas Engelhardt, Nico Kirsch, Aileen Kerfin, Lars P. Lunding, Dominic Ferber, Hannes Buthmann, Ilka Schreier, Carlotta Bosio, Ann‐Kathrin Dobbelstein, Anna Klawonn, Rebecca C. Coll, Lena Bauernhofer, Sandro Keller, Matthias Geyer, Michael Wegmann, Andra B. Schromm, Günther Weindl

**Affiliations:** ^1^ Pharmaceutical Institute, Section Pharmacology and Toxicology University of Bonn Bonn Germany; ^2^ Division of Immunobiophysics, Priority Area Infections, Research Center Borstel, Leibniz Lung Center Member of Leibniz Health Technologies Borstel Germany; ^3^ Division of Lung Immunology, Priority Area Chronic Lung Diseases, Research Center Borstel Leibniz Lung Center Borstel Germany; ^4^ Airway Research North (ARCN) German Center for Lung Research (DZL) Borstel Germany; ^5^ Institute of Structural Biology University of Bonn Bonn Germany; ^6^ Wellcome‐Wolfson Institute for Experimental Medicine Queen's University Belfast Belfast UK; ^7^ Biophysics, Institute of Molecular Biosciences (IMB), NAWI Graz University of Graz Graz Austria; ^8^ Field of Excellence Biohealth University of Graz Graz Austria; ^9^ BioTechMed‐Graz Graz Austria; ^10^ Kiel Nano, Surface and Interface Science KiNSIS Kiel University Kiel Germany

**Keywords:** allergic airway inflammation, asthma, interleukin‐1beta, membrane‐active peptide, NLRP3 inflammasome, Pep19‐2.5

## Abstract

The NLRP3 inflammasome is a multi‐protein complex that plays a crucial role in inflammatory processes mediated by the innate immune system. Dysregulated NLRP3 activation has been implicated in age‐related inflammatory diseases, making it a promising therapeutic target. Here, we report that the synthetic membrane‐active antimicrobial peptide Pep19‐2.5 directly inhibits NLRP3 inflammasome activation. Through cellular, biophysical, and biochemical analyses, we find that Pep19‐2.5 suppresses NLRP3 inflammasome signaling downstream of NLRP3 activation. Pep19‐2.5 interacts with macrophage membranes, supporting a membrane‐targeting mechanism for its anti‐inflammatory effects. Mechanistically, Pep19‐2.5 binds to phosphatidylinositol (PI)‐containing lipid membranes and dispersed trans‐Golgi network (dTGN) structures, which could potentially affect NLRP3 recruitment to the dTGN. We demonstrate a strong and NLRP3‐dependent induction of IL‐1β secretion from human macrophages by house dust mite (HDM) extract, which can be inhibited by Pep19‐2.5. In line with these findings, therapeutic application of Pep19‐2.5 via the nasal aerosol route reduces IL‐1β levels, eosinophil infiltration in bronchoalveolar lavage and significantly improved lung function in an in vivo HDM‐mouse model of allergic airway inflammation. Our findings highlight the therapeutic potential of targeting NLRP3 activation by the small membrane‐active peptide Pep19‐2.5 for the treatment of NLRP3‐driven inflammatory diseases.

## Graphical abstract

The membrane‐active peptide Pep19‐2.5 reduces harmful inflammation by blocking activation of the NLRP3 inflammasome at trans‐Golgi network membranes. By targeting key membrane interactions, Pep19‐2.5 suppresses inflammatory IL‐1β production and alleviates allergic airway inflammation in mice, leading to reduced immune cell infiltration and improved lung function. These findings identify Pep19‐2.5 as a promising candidate for the treatment of NLRP3‐driven inflammatory diseases.

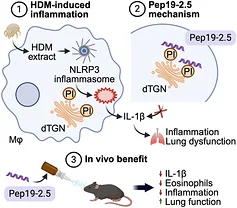

## 1 Introduction

The innate immune system serves as the first line of defense against pathogens. It ensures specificity for danger signals through pattern recognition receptors (PRRs) [1]. These receptors detect pathogen‐associated molecular patterns (PAMPs), triggering immune responses such as inflammation, cellular activation, and recruitment of immune cells [2]. However, excessive activation of the innate immune system can lead to inflammatory disorders, making immune response regulation a key therapeutic target [3]. A major effector of the innate immune system is the pro‐inflammatory cytokine interleukin (IL)‐1β [4]. The secretion of IL‐1β follows a two‐step process involving the priming and activation of the NLRP3 inflammasome [5]. Priming is initiated by the activation of Toll‐like receptors (TLRs) by PAMPs, leading to nuclear translocation of NF‐κB and the expression of *IL1B* and NLRP3‐associated genes. NLRP3 is then activated by the sensing of damage‐associated molecular patterns (DAMPs) such as high extracellular adenosine triphosphate (ATP) concentrations, particulate matter such as monosodium urate (MSU) crystals, or pore‐forming toxins like nigericin [6]. A key factor contributing to these triggers is the efflux of cytosolic potassium ions (K^+^) [7].

Upon activation, NLRP3 undergoes a conformational change from its inactive to active state [8]. Specifically, NLRP3 is recruited to the dispersed *trans*‐Golgi network (dTGN) [9, 10, 11, 12], were it is palmitoylated by zinc finger DHHC domain‐containing S‐acyltransferase 7 (zDHHC7) [13, 14, 15, 16]. Subsequent accumulation of NLRP3 at the dTGN leads to the recruitment and oligomerization of the apoptosis‐associated speck‐like protein containing a caspase recruitment domain (ASC). Following the recruitment and oligomerization of ASC, caspase‐1 is recruited and activated. Caspase‐1 then processes pro‐IL‐1β, pro‐IL‐18, and the pore‐forming pro‐inflammatory protein gasdermin D (GSDMD) into their active secreted forms [17, 18].

The NLRP3 inflammasome has been identified as a key player in a wide range of prevalent diseases, including chronic inflammatory immune disorders and increasingly prevalent lifestyle disorders such as cardiovascular diseases [8, 19]. In response to this urgent medical need, several NLRP3 inhibitors are in clinical and preclinical development. Most of these candidates are designed based on the structure of MCC950 (also known as CRID3). Although MCC950 is highly potent and well‐characterized, it shows adverse effects and limited efficacy against certain NLRP3 mutations [8, 20]. This highlights the need for novel molecules that target NLRP3 activation.

Antimicrobial peptides (AMPs) represent a promising class of anti‐infective therapeutics against antibiotic‐resistant bacteria [21]. Endogenous AMPs, including defensins and cathelicidins, possess broad‐spectrum antibacterial properties, including binding and disrupting pathogen membranes, ultimately causing microbial cell death [22]. A key immunomodulatory mechanism of AMPs is the neutralization of endotoxins such as lipopolysaccharide (LPS) from Gram–negative bacteria, reducing hyperinflammation in sepsis [23, 24]. In addition to directly targeting pathogens or pathogenic factors, AMPs modulate host immune responses, which contributes to their antimicrobial activity [21, 25]. This modulation of immune responses can be mediated by the interaction of AMPs with host cell membranes and contributes to the understanding of their role in inflammatory processes [26]. AMPs can target proteins in the host cell membrane, resulting in impaired function and disturbed membrane formation [27]. NLRP3 membrane association within specific cellular compartments [28, 29], particularly its recruitment to the dTGN, is essential for inflammasome activation and can be blocked pharmacologically by disulfiram [15]. However, the potential influence of AMPs on dTGN in the context of NLRP3 activation has not been investigated.

Previously, we demonstrated that the synthetic LPS‐neutralizing peptide Pep19‐2.5 potently neutralizes bacterial toxins, protects against sepsis in animal models [30], and shows immunomodulatory properties both in vitro and in vivo [31, 32]. Here, we report that Pep19‐2.5 specifically inhibits the NLRP3 inflammasome assembly through a previously unrecognized mechanism of action for NLRP3 inhibitors.

## 2 Results

### 2.1 Pep19‐2.5 Inhibits NLRP3‐Mediated IL‐1β Secretion

Previous studies have demonstrated that LPS‐neutralizing AMPs interact significantly with host cell membranes and inhibit TLR4 activation by changing cholesterol‐containing signaling domains [26]. To explore the role of membrane‐active peptides in the activation of another class of inflammation‐driving innate immune signaling platform—the NLRP3 inflammasome—we pre‐incubated cells with the anti‐LPS peptide Pep19‐2.5 prior to priming and activation. In TLR2‐primed primary monocytes, IL‐1β secretion induced by nigericin (Figure 1A) or MSU crystals (Figure S1) was inhibited in the presence of Pep19‐2.5, with IC_50_ values of 8.0 µM and 12 µM, respectively. Maximum inhibition was observed at 18 µM, with residual IL‐1β secretion of 11% ± 7% for nigericin and 11% ± 1% for MSU, respectively. The effect was confirmed in human monocyte‐derived macrophages (hMDMs), where Pep19‐2.5 inhibited IL‐1β secretion in a concentration‐dependent manner (Figure 1B). Pep19‐2.5 also reduced the secretion of IL‐1β induced by nigericin or MSU crystals in THP‐1 macrophages by 50% and 82%, respectively. (Figure 1C). Pep19‐2.5 induced a slight but statistically significant reduction in metabolic activity only at 18 µM in both THP‐1 macrophages (Figure S2A) and PBMCs (Figure S2B), with cell viabilities of 95% and 73%, respectively. This observation is consistent with previous reports [33, 34] and suggests that Pep19‐2.5 does not exert substantial effects on cell viability at the highest concentration tested. To assess the specificity of Pep19‐2.5 for NLRP3, we evaluated the effects of Pep19‐2.5 on other inflammasome pathways. Pep19‐2.5 showed no inhibitory effect on IL‐1β secretion in primed THP‐1 cells activated with the NLRP1 activator talabostat (Figure 1D) or the AIM2 ligand poly(dA:dT) (Figure 1E), indicating a NLRP3‐specific mode of action for Pep19‐2.5.

**FIGURE 1:**
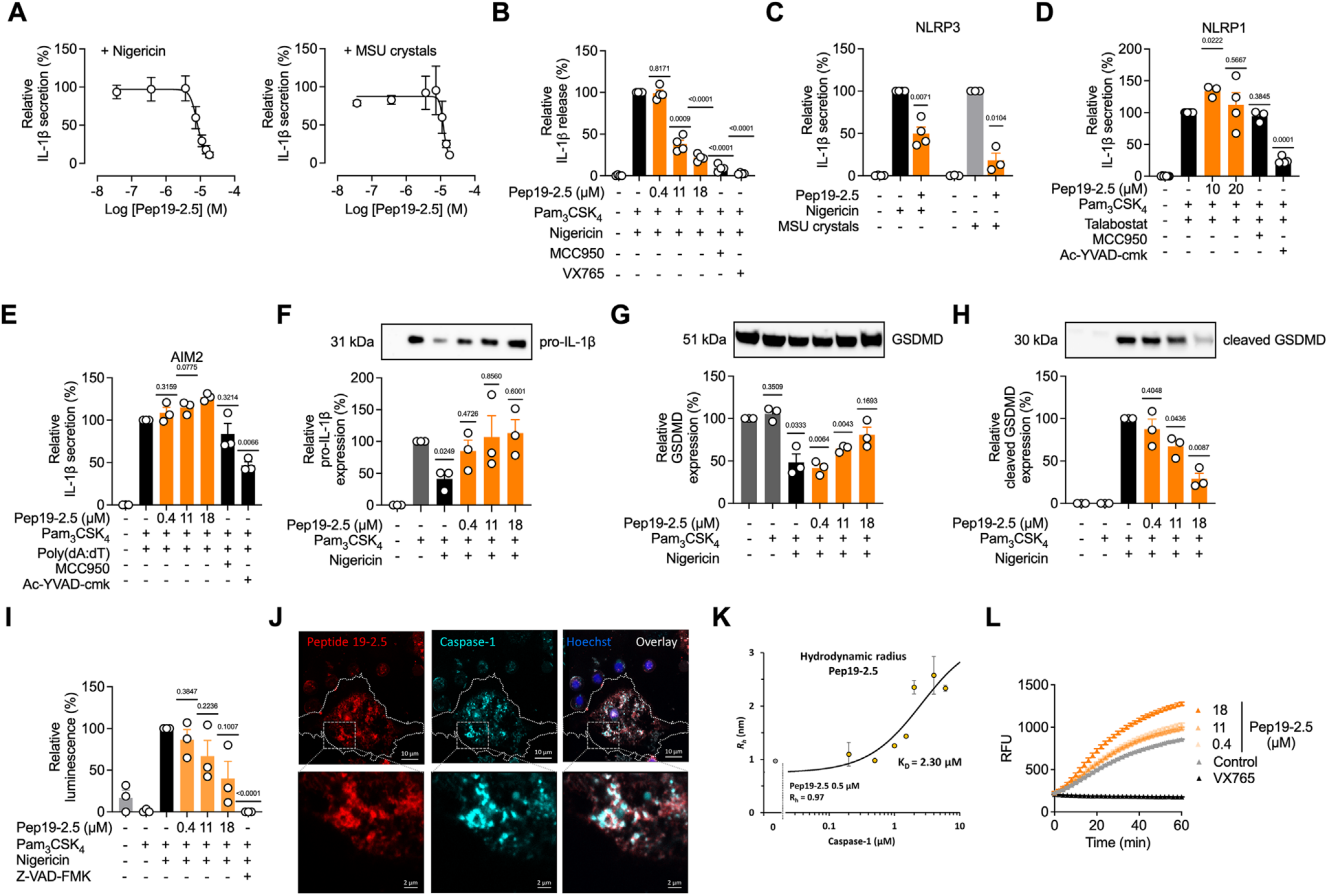
Pre‐incubation with Pep19‐2.5 inhibits nigericin‐ and MSU crystal‐induced IL‐1β secretion. (A) Primary monocytes were primed with 1 µg/mL Pam_3_CSK_4_ for 3 h and then stimulated with 10 µM nigericin or 200 µg/mL MSU crystals for 3 h. Pep19‐2.5 was added 30 min before priming at increasing concentrations ranging from 0.4 to 18 µM. Supernatants were analyzed for IL‐1β by ELISA. Stimulus‐induced IL‐1β secretion was set to 100%. Mean ± SEM (*n*  =  4 biologically independent experiments for nigericin, *n*  =  2 biologically independent experiments for MSU crystals). One‐sample *t*‐test against 100%. (B) hMDMs were primed with 1 µg/mL Pam_3_CSK_4_ for 4 h and then stimulated with 5 µM nigericin for 2 h. Pep19‐2.5 was added at the indicated concentrations 30 min before priming. Supernatants were analyzed for IL‐1β by ELISA. Stimuli‐induced IL‐1β secretion was set to 100%. Mean + SEM (*n*  =  4 biologically independent experiments). One‐sample *t*‐test against 100%. (C) THP‐1 macrophages were primed and stimulated as described in (A). For inhibition experiments, 18 µM Pep19‐2.5 was added 30 min before priming. Supernatants were analyzed for IL‐1β by ELISA. Stimulus‐induced IL‐1β secretion was set to 100%. Mean + SEM (*n*  =  4 biologically independent experiments for nigericin, *n*  =  3 biologically independent experiments for MSU crystals). One‐sample *t*‐test against 100%. (D and E) THP‐1 macrophages were primed and treated with NLRP1 (D) or AIM2 (E) activators. Supernatants were analyzed for IL‐1β by ELISA. Stimulus‐induced IL‐1β secretion was set to 100%. Mean + SEM (*n*  =  3 biologically independent experiments). One‐sample *t*‐test against 100%. (F–H) Protein expression of pro‐IL‐1β (F), full‐length (G), and cleaved GSDMD (H) were analyzed by Western blot. Basal, Pam_3_CSK_4_‐ or nigericin‐induced expression was set to 100%. Mean + SEM (*n*  =  3 biologically independent experiments). One‐sample *t*‐test against 100%. (I) THP‐1 macrophages were primed and stimulated after peptide incubation as described in (A). After 1 h of stimulation with nigericin, the medium was removed, and Z‐WEHD buffer was added according to the manufacturer´s protocol. Ac‐YVAD‐CHO control was subtracted, and stimulus‐induced caspase‐1 activity was normalized to 100%. Mean ± SEM (*n*  =  3 biologically independent experiments). One‐sample *t*‐test against 100%. (J) hMDMs from healthy donors were seeded in µ‐Slides VI and incubated at 37°C in a humidified atmosphere of 5% CO_2_ for 1 h. Macrophages were primed with 1 µg/mL Pam_3_CSK_4_ for 3 h and stimulated with 10 µM nigericin for 1 h in the presence of fluorophore‐conjugated Rh‐Pep19‐2.5. Active caspase‐1 was stained with the caspase‐1 pseudosubstrate FLICA660‐YVAD‐FMK. Cells were washed and fixed, and nuclei were stained with Hoechst 34580. Confocal microscopy images are representative of *n*  =  2 biologically independent experiments. Scale bar is 10 µm (upper panel) and 2 µm (zoomed in lower panel). (K) Solutions containing 0.5 µM Atto488‐conjugated Pep19‐2.5 were prepared in the presence of increasing concentrations of recombinant human caspase‐1 and incubated overnight at 4°C under gentle agitation. Microfluidic diffusional sizing (MDS) measurements were performed at room temperature to determine the hydrodynamic radius (*R*_h_) of fluorescently labeled particles. Dots represent the mean ± SD of replicates (*n* ≥ 3) of free peptide and peptide–protein complexes, and the black line represents the best nonlinear fit according to Equation 1. (L) Human recombinant caspase‐1 was incubated with the caspase‐1‐specific substrate Ac‐YVAD‐AMC at the indicated concentrations of Pep19‐2.5 or 40 µM of the caspase‐1 inhibitor VX765. Enzyme activity in relative fluorescence units (RFU) was measured every 2 min at 37°C. Representative traces are means ± SEM (*n*  =  2 biologically independent experiments).

Next, we investigated whether Pep19‐2.5 inhibits the NLRP3 inflammasome pathway upstream of IL‐1β secretion. Caspase‐1 activation by NLRP3 is characterized by self‐cleavage of pro‐caspase‐1, thereby forming active caspase‐1 [35]. Stimulation of primed THP‐1 macrophages with nigericin induced a robust pro‐IL‐1β expression, which was reduced when NLRP3 was activated by nigericin. Incubation with increasing concentrations of Pep19‐2.5 resulted in a recovery of pro‐IL‐1β at all concentrations tested (Figure 1F). Incubation with Pep19‐2.5 also recovered the caspase‐1 substrate GSDMD (Figure 1G) and reduced the amount of cleaved GSDMD (Figure 1H). Furthermore, Pep19‐2.5 concentration‐dependently decreased caspase‐1 activity, although this effect did not reach statistical significance (Figure 1I). To evaluate the direct effect of Pep19‐2.5 on caspase‐1, we examined the intracellular staining of caspase‐1 enzymatic activity using a caspase‐1‐specific FLICA substrate in nigericin‐stimulated human macrophages (Figure 1J). Activated caspase‐1 was surrounded by Pep19‐2.5‐positive membranous compartments, demonstrating a tight spatial association between the peptide and active caspase‐1 (Figure 1J, enlarged areas). Microfluidic diffusional sizing (MDS) revealed binding of Atto488‐conjugated Pep19‐2.5 to recombinant human caspase‐1 with a dissociation constant (*K*_D_) of 2.3 µM (Figure 1K). However, functional studies with recombinant human caspase‐1 revealed that Pep19‐2.5 does not inhibit caspase‐1 enzyme activity in vitro (Figure 1L), indicating that Pep19‐2.5 acts upstream of caspase‐1 substrate processing.

### 2.2 Pep19‐2.5 Does Not Affect Priming or Interfere with the NLRP3 Activator Nigericin

We selected the TLR2 agonist Pam_3_CSK_4_ as the priming stimulus to avoid potential interference of the LPS‐neutralizing peptide with TLR4 priming in the presence of LPS. Binding studies based on microfluidic diffusional sizing (MDS, Figure S3A) and isothermal titration calorimetry (ITC) [36] indicate that Pep19‐2.5 and Pam_3_CSK_4_ do not interact. To exclude negative regulation of Pep19‐2.5 on TLR2‐mediated priming in our cell stimulation experiments, we analyzed cytokine expression upon priming. In THP‐1 macrophages, Pam_3_CSK_4_ induced pro‐IL‐1β expression, whereas pre‐incubation with Pep19‐2.5 did not alter protein levels (Figure 2A). This was confirmed at the transcriptional level in THP‐1 macrophages (Figure S3B) and primary monocytes (Figure S3C), where no significant concentration‐dependent regulation of *IL1B* and *TNF* expression was observed.

**FIGURE 2:**
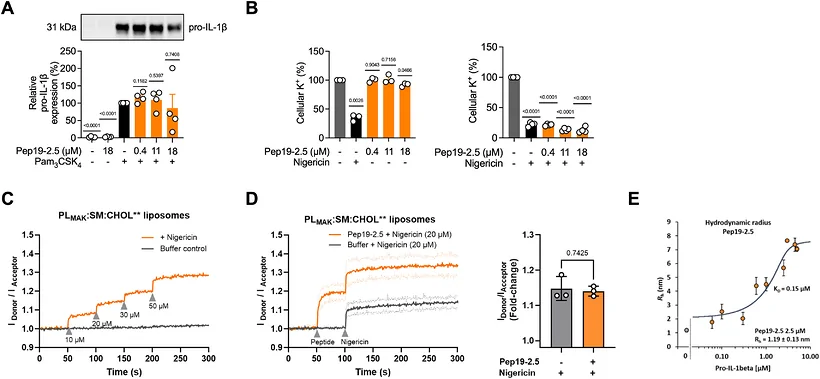
Pep19‐2.5 does not interfere with Pam_3_CSK_4_‐mediated priming or nigericin. (A) THP‐1 macrophages were primed with 1 µg/mL Pam_3_CSK_4_ for 3 h. Pep19‐2.5 was added 30 min before priming at increasing concentrations ranging from 0.4 to 18 µM. Protein expression of pro‐IL‐1β (31 kDa) was analyzed by Western blot. Pam_3_CSK_4_‐induced expression was set to 100%. Mean + SEM (*n*  =  4 biologically independent experiments). One‐sample *t*‐test against 100%. (B) THP‐1 macrophages were incubated with 10 µM nigericin or indicated concentrations of Pep19‐2.5 for 30 min (left panel). For combination experiments, Pep19‐2.5 was added 30 min before the addition of nigericin (right panel). Cellular K^+^ content was determined by AAS. Absorption of control was set to 100%. Mean + SEM (*n*  = 3 biologically independent experiments, *n*  =  4 biologically independent experiments for combination experiments). One‐sample *t*‐test against 100%. (C and D) Human macrophage model membrane liposomes PL_MAK_:SM:Chol (2:0.5:0.2 M) containing fluorophore‐conjugated phospholipids *NBD‐PE, donor, and *Rh‐DHPE, acceptor, were analyzed by Förster‐resonance‐energy‐transfer in a probe‐dilution assay at a physiological temperature of 37°C. Signals were recorded for 50 s to determine the baseline, then nigericin was added every 50 s at the indicated final concentrations. Fluorescence intensity ratios I_Donor_/I_Acceptor_ were calculated. Lines show the mean of *n*  =  3 biologically independent measurements. (D) Liposomes, as in (C), were recorded for base line signal at 37°C. Pep19‐2.5 (20 µM) was added at *t*  =  50 s and allowed to intercalate into the liposome membrane. At *t*  =  100 s nigericin (20 µM) was added under constant stirring. Fold‐change induced by nigericin in the absence and presence of Pep19‐2.5 at *t*  =  300 s was calculated, and statistical significance was analyzed by unpaired *t*‐test. Lines show the mean, dotted lines SD of *n*  =  3 biologically independent measurements. (E) Solutions containing 0.5 µM rhodamine‐conjugated Pep19‐2.5 were prepared in the presence of increasing concentrations of recombinant human pro‐IL‐1β and incubated overnight at 4°C under gentle agitation. Microfluidic diffusional sizing (MDS) measurements were performed at room temperature to determine the hydrodynamic radius (*R*_h_) of fluorescently labeled particles. Dots represent the mean ± SD of replicates (*n* ≥ 3) of free peptide and peptide–protein complexes and the black line represents the best nonlinear fit according to Equation (1).

To gain insights into the initial stage of inflammasome activation, we investigated the effect of Pep19‐2.5 on the NLRP3 activator nigericin. Nigericin‐induced K^+^ efflux is a common trigger of NLRP3 activation [37]. It has been hypothesized that nigericin acts as an ionophore by shuttling K^+^ ions across phospholipid membranes [38]. Indeed, stimulation of primed THP‐1 macrophages with 10 µM nigericin resulted in a strong and significant decrease by 65% ± 3.3% in intracellular K^+^, as determined by atomic absorption spectroscopy (AAS) [39] (Figure 2B). In contrast, Pep19‐2.5 alone resulted only in a slight decrease of 8.0% ± 1.8% in intracellular K^+^ at the highest concentration (18 µM Pep19‐2.5). Incubation of THP‐1 macrophages with Pep19‐2.5 before the addition of nigericin failed to restore intracellular K^+^ (Figure 2B). The incubation of macrophage model‐membrane liposomes with increasing concentrations of nigericin induced a sustained increase in liposome surface area, thereby showing membrane insertion (Figure 2C). Pre‐incubation with Pep19‐2.5 did not inhibit the surface area increase observed for nigericin (Figure 2D).

As electrostatic interactions are critical for pro‐IL‐18 and presumably also for pro‐IL‐1β processing [40], we investigated binding of the basic Pep19‐2.5 peptide to the negatively charged pro‐IL‐1β protein [41]. MDS experiments revealed a concentration‐dependent increase in the apparent hydrodynamic radius (*R*_h_) of Pep19‐2.5 (Figure 2E), demonstrating pro‐IL‐1β complex formation with a dissociation constant (*K*_D_) of 0.15 µM. However, this interaction does not lead to reduced pro‐IL‐1β expression (Figure 2A), suggesting that direct sequestration or degradation of pro‐IL‐1β is unlikely to account for the inhibitory effect of Pep19‐2.5 on IL‐1β secretion. Further biophysical and functional studies are needed to evaluate the potential impact of Pep19‐2.5 binding to pro‐IL‐1β.

### 2.3 Pep19‐2.5 Interacts with Host Cell Membranes and Modulates Membrane Fluidity

Certain LPS‐neutralizing peptides, specifically the human cathelicidin LL‐37 and polymyxin B, are known to interact with host cell membranes, which is a crucial part of their multimodal mode of action [26]. The inhibitory effect of Pep19‐2.5 observed for nigericin‐ and MSU crystal‐induced IL‐1β secretion persisted even after washing the cells and removing Pep19‐2.5 from the supernatant (Figure 3A), indicating that Pep19‐2.5 exerts its effects either intracellularly or through a partly membrane‐dependent mechanism. To better define the membrane‐interaction properties of Pep19‐2.5, we assessed how Pep19‐2.5 and two derivative peptides—Pep19‐2.5gek (a truncation variant) and Pep19‐2.5KO (a scrambled control) (Figure 3B)—interact with model membranes, which proved to be an important tool in deciphering the mode of action of LL‐37 and polymyxin B [26]. The incubation of macrophage model‐membrane liposomes with Pep19‐2.5 and its variants Pep19‐2.5KO and Pep19‐2.5gek resulted in a significantly increased membrane surface area at the lowest concentration tested (0.125 µM) compared to vehicle control, as determined by FRET measurements (Figure 3C). While incubation with increasing concentrations of Pep19‐2.5gek barely further increased the surface area at the highest concentrations tested, Pep19‐2.5 and Pep19‐2.5KO continued to robustly increase the surface area at all concentrations tested. To further elucidate the membrane interactions of Pep19‐2.5 and its variants, we assessed changes in model‐membrane fluidity using the fluorescent probe 1,6‐diphenyl‐1,3,5‐hexatriene (DPH) [42]. All three peptides significantly increased fluorescence polarization (FP), indicating a reduction in the membrane fluidity immediately after addition, while only Pep19‐2.5 was able to induce a sustained rigidization of the membrane over the entire recording period of 250 s (Figure 3D). This observation was consistent with data obtained from human macrophages. Again, only Pep19‐2.5, but neither Pep19‐2.5KO nor Pep19‐2.5gek, induced a sustained membrane rigidization (Figure 3E). In line with these data, Pep19‐2.5KO did not reduce nigericin‐induced IL‐1β release, and Pep19‐2.5gek showed only a modest inhibitory effect, which was noticeably weaker than that of Pep19‐2.5 (Figure 3F). These observations led us to hypothesize that Pep19‐2.5 may interfere with IL‐1β‐releasing mechanisms. To test this hypothesis, we examined the intracellular enrichment of IL‐1β but observed no significant effects (Figure S4).

**FIGURE 3:**
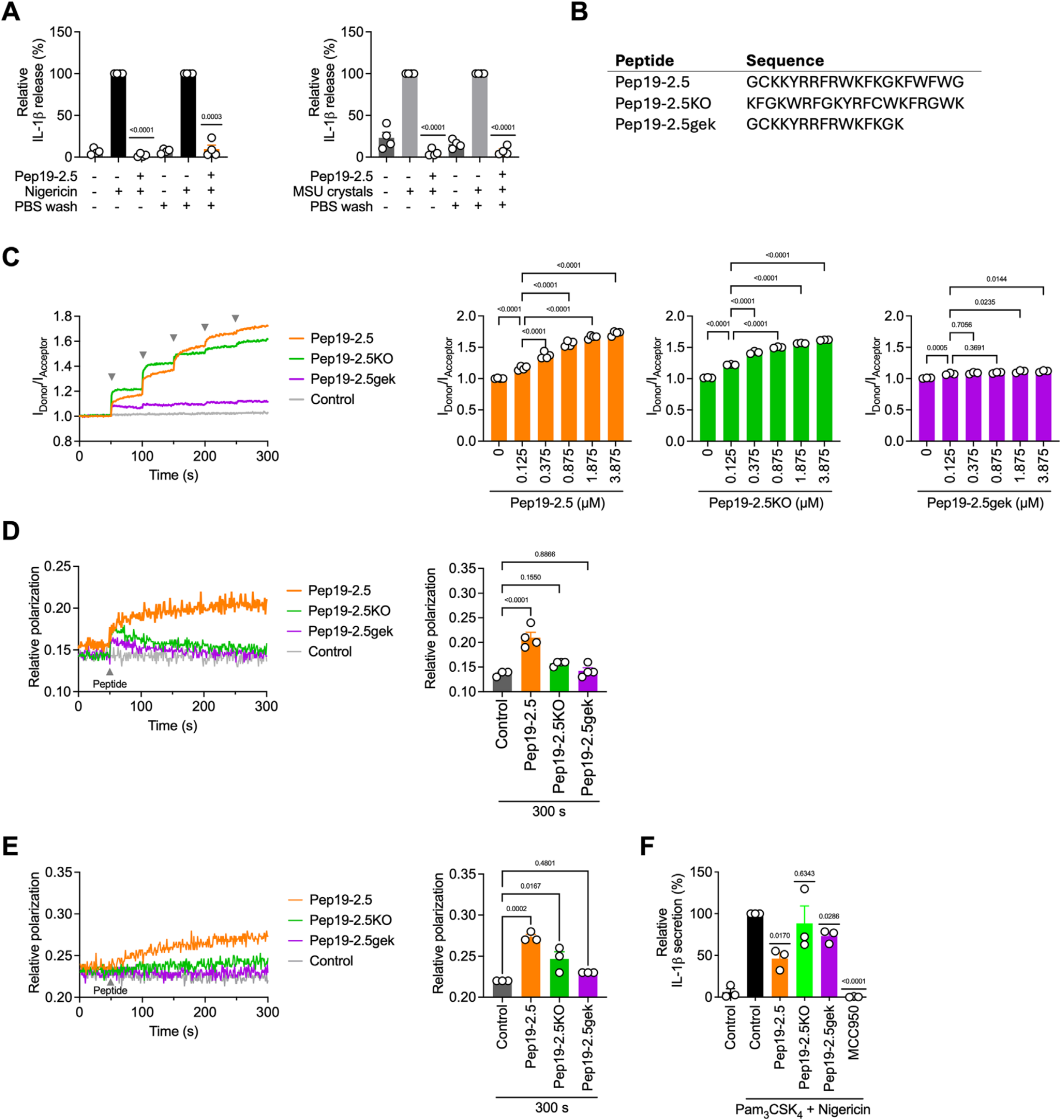
Membrane interactions of Pep19‐2.5. (A) Primary monocytes were primed with 1 µg/mL Pam_3_CSK_4_ for 3 h and then stimulated with 10 µM nigericin or 200 µg/mL MSU crystals. Pep19‐2.5 (18 µM) was added 30 min before priming. Where indicated, cells were washed twice with PBS after incubation with Pep19‐2.5. Supernatants were analyzed for IL‐1β by ELISA. Stimulus‐induced IL‐1β secretion was set to 100%. Bar graphs show Mean + SEM (*n*  =  4 biologically independent experiments). One‐sample *t*‐test against 100%. (B) Amino acid sequences of Pep19‐2.5, Pep19‐2.5KO, and Pep19‐2.5gek. (C) Changes in the membrane surface of macrophage model membrane liposomes were analyzed by a Förster‐resonance‐energy‐transfer (FRET)‐based assay. PLMAK:SM:Chol (2:0.5:0.2 M) liposomes containing *NBD‐PE (donor) and *Rh‐DHPE (acceptor) were diluted to 10 µM. Peptides were added at the indicated final concentrations and signals were recorded for 50 s after each titration step. Fluorescence intensity ratios I_Donor_/I_Acceptor_ were calculated. Curves show mean, and bar graphs show mean + SEM (*n*  =  3 independent experiments except Pep19‐2.5 *n*  =  4). One‐way ANOVA followed by Dunnett's post‐test. (D) Macrophage model membrane liposomes PLMAK:SM:Chol (2:0.5:0.2 M) were labeled with DPH. Changes in membrane fluidity were determined by relative polarization of the fluorescence emission of DPH. Peptides were added in a concentration of 3.875 µM at the indicated timepoint. The measurement was performed over 250 s at a constant temperature of 37°C. Curves show mean, and bar graphs show mean + SEM (*n*  =  4 independent experiments except control *n*  =  3). One‐way ANOVA followed by Dunnett's post‐test. (E) Human macrophages of healthy donors were membrane‐labeled with DPH. Changes in membrane fluidity were determined by relative polarization of the fluorescence emission of DPH at 37°C. Peptides were added at time point 50 s to a final concentration of 3.875 µM. Curves show mean, bar graphs show mean + SEM at indicated time points (*n*  =  3 independent measurements on cells from one donor; data representative for *n*  =  4 individual donors. One‐way ANOVA followed by Dunnett's post‐test. (F) Primary monocytes were primed with 1 µg/mL Pam_3_CSK_4_ for 3 h and then stimulated with 10 µM nigericin for 3 h. 7 µM of the Pep19 variants were added 30 min before priming. Supernatants were analyzed for IL‐1β by ELISA. Nigericin‐induced IL‐1β secretion was set to 100%. Mean + SEM (*n * =  3 biologically independent experiments). One‐sample *t*‐test against 100%.

### 2.4 Pep19‐2.5 Inhibits NLRP3 Inflammasome Assembly and IL‐1β Processing

To further clarify the inhibitory mechanism of Pep19‐2.5 on NLRP3‐mediated IL‐1β secretion, we studied the activation of the NLRP3 inflammasome. Following K^+^ efflux, NLRP3 undergoes a conformational change, transitioning from an inactive to an active conformation [8]. Thereafter, active NLRP3 recruits ASC, which can be observed by monitoring ASC oligomerization and ASC speck formation [43]. We covalently crosslinked THP‐1 cell pellets and isolated ASC oligomers. Pam_3_CSK_4_ and nigericin strongly induced ASC oligomerization, which was inhibited by Pep19‐2.5 in a concentration‐dependent manner (Figure 4A). Likewise, the downstream formation of NLRP3‐ASC specks, assessed in HEK293^ASC‐BFP^ cells transduced with NLRP3, was also inhibited by Pep19‐2.5 in a concentration‐dependent manner (Figure 4B). In PMA‐differentiated THP‐1^C1C‐EGFP^ reporter cells [44], Pep19‐2.5 reduced Pam_3_CSK_4_‐ and nigericin‐induced caspase‐1^CARD^ (C1C) and ASC speck formation, as observed by confocal microscopy (Figure 4C) and confirmed by quantitative analysis (Figure 4D). This effect was consistent across the reporter readouts, which showed fewer specking cells with increasing peptide concentrations.

**FIGURE 4:**
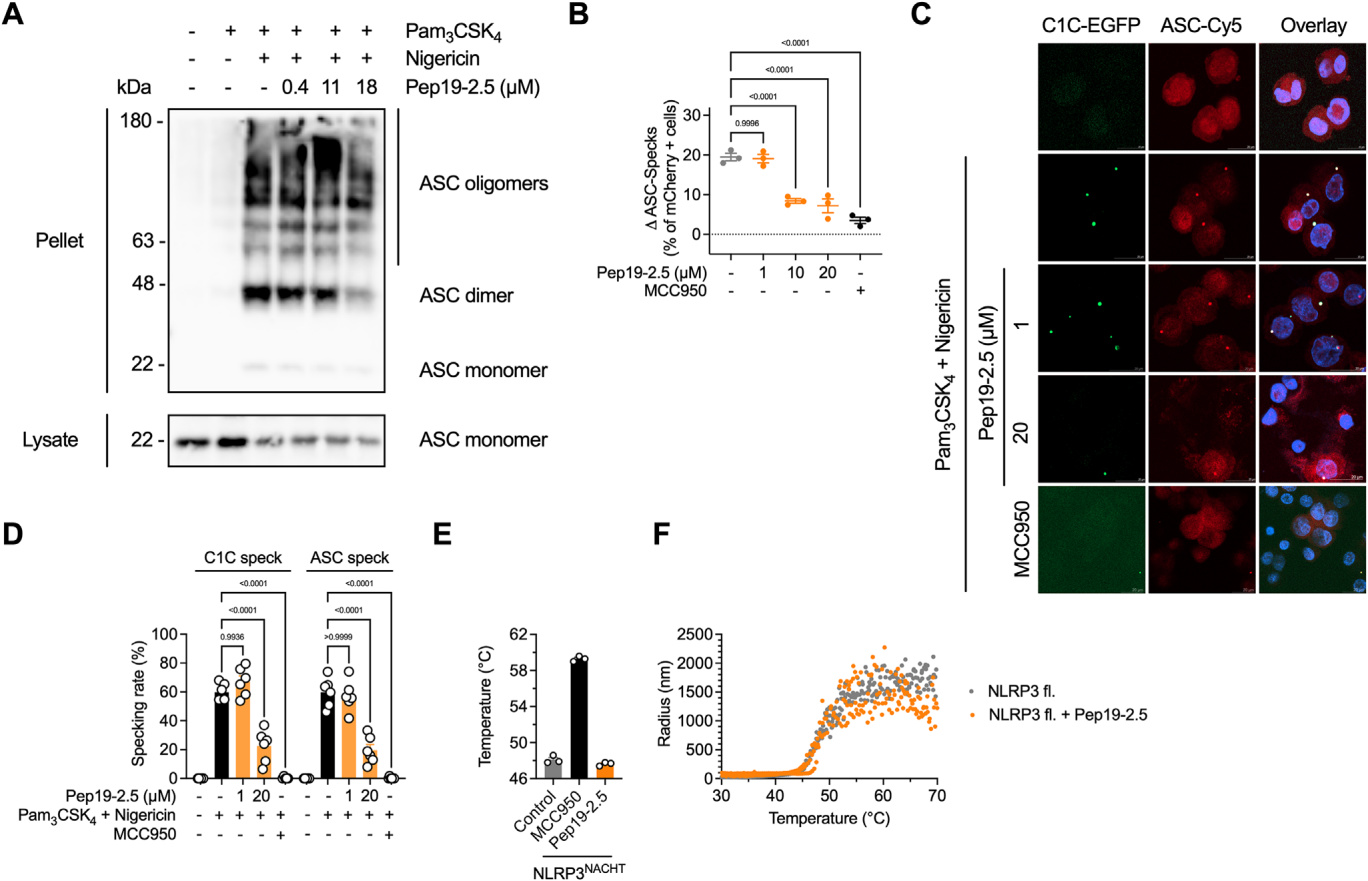
Pep19‐2.5 inhibits NLRP3‐induced ASC assembly but does not bind to NLRP3. (A) Representative Western blot of cross‐linked cytosolic pellet from primed THP‐1 macrophages that were pre‐incubated with increasing concentrations of Pep19‐2.5 and stimulated with 10 µM nigericin for 1 h (*n*  =  3 biologically independent experiments). (B) HEK293^ASC‐BFP^ cells transduced with NLRP3 were pre‐incubated with increasing concentrations of Pep19‐2.5 or MCC950 and stimulated with nigericin. ASC speck formation was determined by flow cytometry. Dot plots show mean ± SEM (*n*  =  3 biologically independent experiments). One‐way ANOVA followed by Dunnet's post‐test. (C) THP‐1^C1C‐EGFP^ macrophages were incubated with the indicated concentrations of Pep19‐2.5 for 30 min prior to priming. After priming with Pam_3_CSK_4_, 40 µM VX765 was added to limit cell death, and 10 µM nigericin was added to stimulate NLRP3 assembly for 1 h. Staining was performed using an anti‐ASC antibody and Hoechst 34580. Confocal microscopy images are representative of *n*  =  2 biologically independent experiments, with three individual images taken for each condition. Scale bar  =  20 µm. Full images are shown Figure S5. (D) Nuclei, ASC, and C1C specks in the images shown in (C) were quantified separately in the corresponding fluorescence channels. For each condition, three independent microscopy images were analyzed, with each data point representing a single image. Speck formation was quantified manually, and results are presented as mean + SEM. One‐way ANOVA followed by Šídák's multiple comparison post hoc analysis. (E) Melting temperature of NLRP3^NACHT^ alone, with the inhibitor MCC950 or Pep19‐2.5, as determined by nanoDSF (*n*  =  3 independent experiments). No shift upon addition of Pep19‐2.5 is observed, indicating no direct interaction of Pep19‐2.5 with the NACHT domain of NLRP3. (F) The thermal stability of the decameric NLRP3 (fl., wt) protein with and without Pep19‐2.5 was determined by measuring the hydrodynamic radius by DLS (*n*  =  3 independent experiments).

To evaluate whether Pep19‐2.5 directly interacts with NLRP3, we examined the interaction between Pep19‐2.5 and the NACHT domain of NLRP3 (amino acids 131–694) [45]. While a 3.3‐fold excess of the inhibitor MCC950 increased the thermal stability of NLRP3 by approximately 18°C compared to the DMSO control, Pep19‐2.5 caused no change in the melting temperature (Figure 4C). Furthermore, dynamic light scattering (DLS) analysis using full‐length NLRP3 in the ADP‐bound form showed no alteration of the disassembly of the decameric state, measuring the hydrodynamic radius upon temperature increase (Figure 4D). From these experiments, we conclude that there is no direct interaction between NLRP3 and Pep19‐2.5.

### 2.5 Pep19‐2.5 Associates With PI‐Lipid‐Containing Membranes and dTGN Structures

Association of NLRP3 with the dTGN is a critical accelerator of inflammasome assembly involving specific lipid interaction. Especially a polybasic region including a KMKK motif of lysine residues (amino acids 131–134) and another positively charged region (amino acids 135–147) between the pyrin domain and the NACHT domain of NLRP3 mediate docking of cytosolic NLRP3 to the TGN‐specific phosphatidylinositol (PI)‐species phosphatidyinositol‐4‐phosphate (PI4P) [9]. The dTGN thereby acts as a scaffold, inducing clustering of NLRP3 and palmitoylation of NLRP3 by dTGN‐localized ZDHHC7, supporting oligomer formation of membranous NLRP3 and eventually enhanced aggregation to NLRP3 inflammasome complexes [9, 13, 14]. Thus, docking of cytosolic NLRP3 to PI‐lipid species at the dTGN is a crucial step in inflammasome assembly, and disruption of this interaction blocks NLRP3 aggregation [9].

A comparison of Pep19‐2.5 and human NLRP3 identified a region of notable sequence similarity corresponding to amino acids 129–148 in NLRP3. AlphaFold models indicate remarkable structural similarity with the amphipathic organization of the peptides (Figure 5A,B). This prompted us to investigate the association of the polycationic peptide Pep19‐2.5 with PI‐containing membranes in liposome assays. Electrophoretic light scattering (ELS) analysis revealed a concentration‐dependent reduction of the membrane zeta‐potential by Pep19‐2.5 in liposomes containing 5 mol% PI3P, characteristic of endosomal membranes, or 5 mol% PI4P, characteristic of dTGN‐membranes (Figure 5C). Peptide binding manifested in a strong reduction of the zeta‐potential with charge neutralization around 0.5 µM peptide and over‐compensation at higher peptide concentrations. Peptide binding was also observed to DOPC liposomes, indicating contributions of hydrophobic and electrostatic components to the membrane interactions of Pep19‐2.5. In contrast, NLRP3 (129–148), resembling the dTGN interacting domain, bound to PI3P and PI4P containing membranes, but it did not induce over‐compensation of the surface charge and bound only weakly to neutral DOPC membranes (Figure 5D).

**FIGURE 5:**
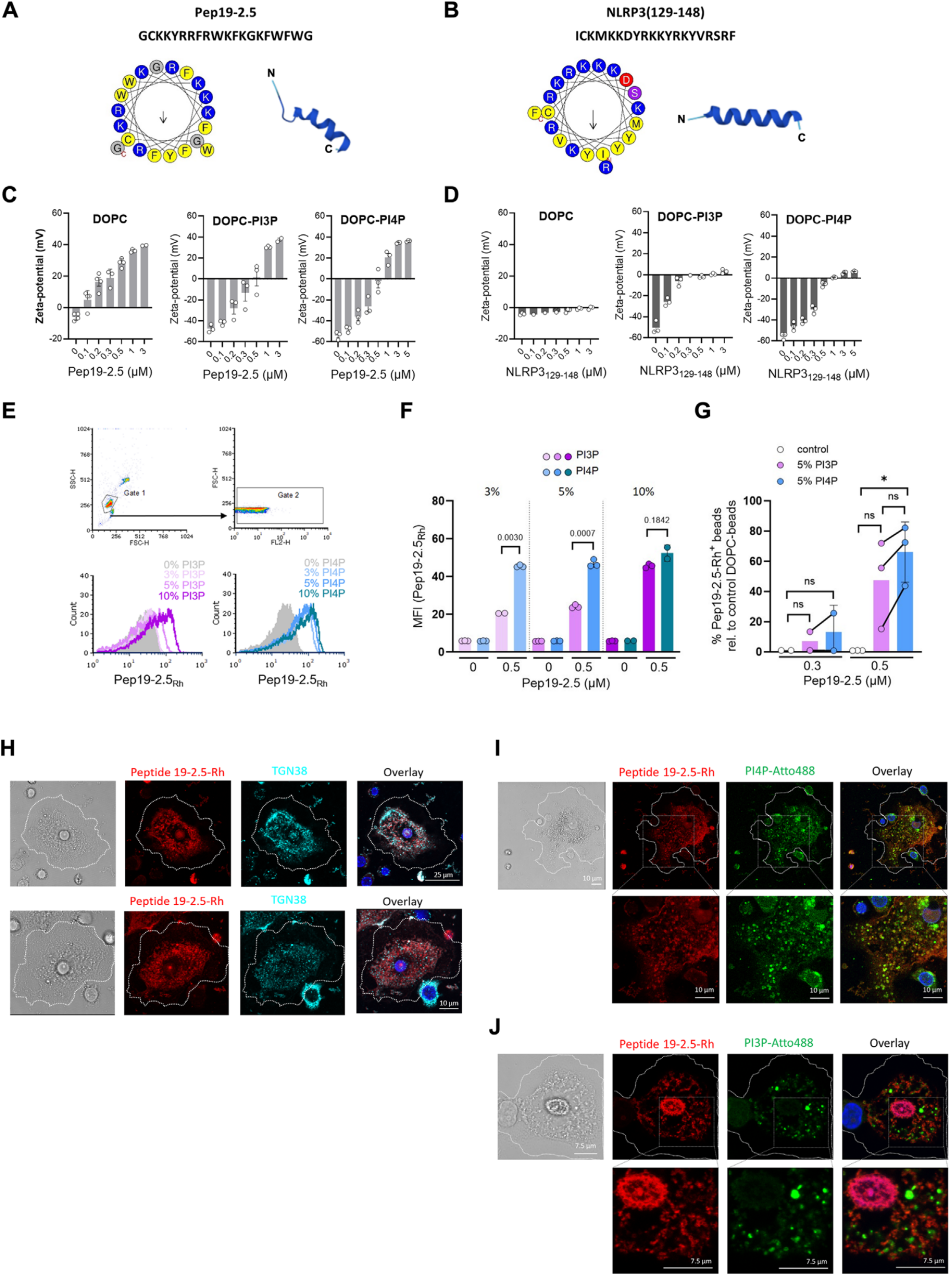
Pep19‐2.5 associates with PI‐lipid‐containing membranes and TGN structures. (A) Amino acid sequences of Pep19‐2.5 and (B) NLRP3 (amino acids 129–148), and their predicted structures generated using AlphaFold3 [47]. Helical wheel projections were created using HeliQuest (http://heliquest.ipmc.cnrs.fr/cgi‐bin/ComputParamsV2.py). In the helical wheel diagrams, hydrophobic residues are shown in yellow, arginine and lysine in blue, serine in purple, aspartate in red, and glycine in gray. Arrows indicate the direction and magnitude of the hydrophobic moment. (C) Pep‐19‐2.5 or (D) NLRP3 peptide was titrated at the indicated final concentrations to liposomes made from DOPC, DOPC‐PI3P, and DOPC‐PI4P and the zeta potential of the liposomes was determined at 25°C by ELS in triplicate measurements for each concentration. Data shown are mean and SEM of *n*  =  3 independent titration experiments. (E) Silica beads were coated with DOPC membranes containing 0, 3, 5, and 10 mol% PI3P or PI4P. Membrane‐coated beads (MCBs) were incubated with 0.5 µM rhodamine‐conjugated Pep‐19‐2.5‐Rh, washed, gated and analyzed by flow cytometry. (F) Mean fluorescence intensities (MFIs) of triplicate measurements of a representative experiment out of *n*  =  3 independent experiments. (G) Fraction of Pep19‐2.5‐Rh‐positive beads from MCB containing 5 mol% PI3P or PI4P. Data were normalized to DOPC MCB. Data from *n*  =  3 independent experiments. (H) hMDMs from healthy donors were seeded in µ‐Slides VI and incubated at 37°C in a humidified atmosphere of 5% CO_2_ for 1 h. After medium change, macrophages were exposed to 18 µM Pep19‐2.5‐Rh for 30 min at 37°C, washed three times with ice‐cold PBS, fixed, permeabilized, and stained with primary antibody to dTGN (TGN38/TGOLN2) and secondary antibody Alexa647‐conjugated goat anti‐mouse IgG (H+L). Cells were washed, fixed, and nuclei stained with Hoechst 34580. Cells were analyzed on a Leica TCS SP5 confocal laser scanning microscope equipped with hybrid HyD detectors. Confocal microscopy images are representative of *n*  =  3 biologically independent experiments. Scale bar  =  25 and 10 µm. (I and J) hMDMs from healthy donors were exposed to Rh‐Pep19‐2.5 for 30 min at 37°C as described in (H), washed, fixed, quenched, permeabilized, and stained with Atto488‐conjugated biosensors [46] to (I) PI3P or (J) PI4P. Cells were washed, fixed, and nuclei were stained with Hoechst 34580. Confocal microscopy images are representative of *n*  =  3 biologically independent experiments. Scale bar  =  10 µm and 7.5 µm.

Pep19‐2.5 binding was investigated in more detail by flow cytometry analysis of membrane‐coated beads (MCBs), allowing sensitive detection and quantification. MCBs were gated (Figure 5E) and PI lipid species were confirmed by staining with PI3P‐ and PI4P‐specific biosensors [46] (Figure S6). Mean fluorescence intensities of beads incubated with Pep19‐2.5 increased with PI3P or PI4P content in MCB (Figure 5E) and showed significantly higher binding of fluorophore‐conjugated Pep19‐2.5 to 3 mol% and 5 mol% PI4P‐containing membranes than to PI3P‐containing membranes (Figure 5F). Also, the fraction of Pep19‐2.5 positive beads was larger for PI4P‐ than for PI3P‐containing membranes (Figure 5G). Confocal microscopy experiments on unstimulated human macrophages demonstrate that Pep19‐2.5 is rapidly taken up, localizes to intracellular membranes, and accumulates in perinuclear compartments, suggesting potential dTGN localization (Figure 5H). Staining human macrophages confirmed TGN localization as shown by partial colocalization with TGN marker protein (Figure 5H) and the TGN specific membrane lipid PI4P (Figure 5I). In contrast, Pep19‐2.5 did not colocalize with the endosomal marker lipid PI3P (Figure 5J). These data demonstrate enhanced attraction of Pep19‐2.5 to PI4P‐containing model membranes and to PIP4 of the TGN in human macrophages, identifying PI4P as a molecular trait and the mechanistic target of Pep19‐2.5.

### 2.6 Pep19‐2.5 Reduces Pro‐Inflammatory IL‐1β Response to House Dust Mite Extract In Vitro and In Vivo

As a central regulator of inflammation, it is not surprising that NLRP3 inflammasome activation and IL‐1 cytokines have been associated with numerous chronic inflammatory diseases. In addition to Crohn's disease, arteriosclerosis, gout, and Alzheimer's disease, bronchial asthma represents one of the most important chronic diseases, affecting more than 260 million people worldwide [48]. Despite the introduction of several therapeutic antibodies such as dupilumab or omalizumab, standard treatment of asthma patients still relies on corticosteroids (CS), which are associated with serious side effects when applied systemically [49]. Especially severe or uncontrolled asthma phenotypes, type‐2‐low asthma endotypes, as well as acute asthma exacerbations, are often associated with considerable CS refractoriness so that treatment options for affected patients represent an unmet medical need [50]. House dust mite (HDM) is one of the most common aeroallergens worldwide to which up to 85% of all asthma patients are allergic [51]. We have used a well‐established mouse model of HDM‐induced experimental allergic asthma (EEA) to demonstrate that both the NLRP3 inflammasome and IL‐1β are critically important for the pathogenesis of the disease and also represent a promising target for therapeutic intervention [52, 53].

To evaluate the role of HDM in macrophage inflammasome activation, unprimed human macrophages from healthy donors were stimulated with HDM extract. These experiments established that HDM induces a robust concentration‐dependent IL‐1β production (Figure 6A). This response is significantly inhibited by the NLRP3‐specific inhibitor MCC950 (Figure 6B), indicating a primary dependence on NLRP3 inflammasome activation in the HDM‐induced IL‐1β production by macrophages. The TLR4‐specific antagonist eritoran did not inhibit IL‐1β production (Figure 6C), excluding involvement of a potential endotoxin contamination of the HDM extract. Pep19‐2.5 significantly inhibited HDM‐induced IL‐1β release at 20 µM concentration at different concentrations of HDM (Figure 6D–F).

**FIGURE 6:**
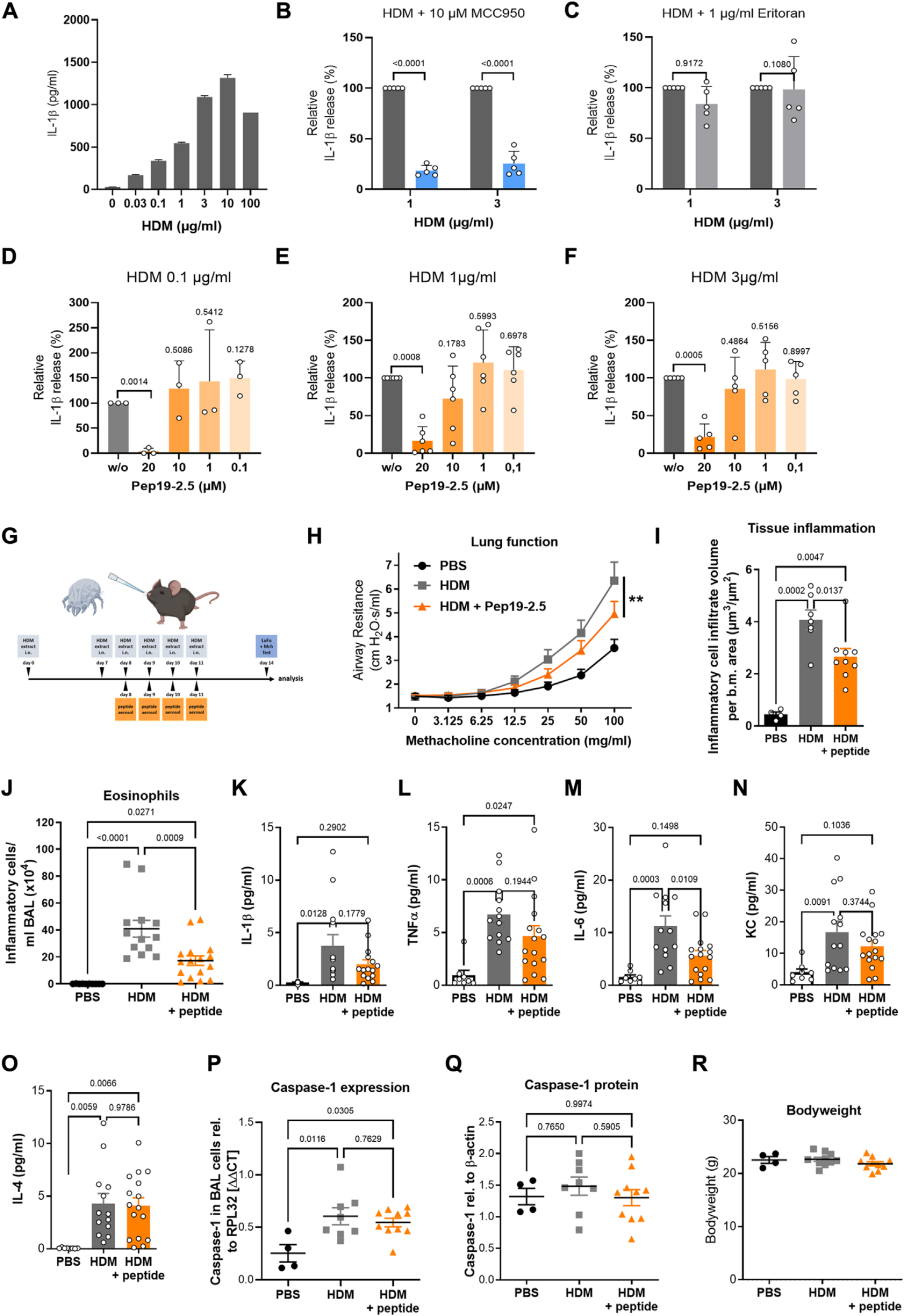
Pep19‐2.5 reduces pro‐inflammatory IL‐1β response to house dust mite extract in vitro and in vivo. (A–E) Human macrophages from heathy donors were seeded in serum‐free Opti‐MEM medium and stimulated without priming with the indicated doses of house dust mite (HDM) extract for 24 h at 37°C (A) alone, in the presence of (B) the NLRP3 inhibitor MCC950 (10 µM), (C) the TLR4 antagonist eritoran (1 µg/mL), or (D–F) Pep19‐2.5. IL‐1β release was determined from cell‐free supernatants by ELISA. Data shown in (A) mean + SD of duplicate analyses and are representative for *n*  =  7 independent donors. Data in (B–E) were normalized to 100% for HDM in the absence of inhibitors and are shown as mean + SEM of (B, C, E, F): *n*  =  5 and (D): *n*  =  3 independent experiments with cells from different healthy donors. Statistical significance against HDM alone was analyzed by a two‐sided *t*‐test. (G) Treatment protocol for HDM‐induced allergic asthma mouse model. (H) Airway resistance in response to methacholine inhalation, (I) inflammatory cell infiltrate volume in lung tissue per epithelial basal membrane (b.m.) area, and (J) numbers of eosinophils on day 14 in bronchoalveolar lavage (BAL) fluid of healthy (PBS), asthmatic (HDM), or Pep19‐2.5‐treated mice (HDM + peptide), *n*  = 10 mice per group. (K–O) Cytokine levels in BAL fluid were assessed by MSD U‐Plex assays. (P) Caspase‐1 mRNA expression in BAL cells and (Q) caspase‐1 protein determined by Western blot. (R) Final body weight on day 14 of the treatment protocol. Results are presented as mean values ± SEM. Statistical significance was assessed using ordinary one‐way ANOVA and Tukey´s multiple comparison post hoc analyses.

To test the pathophysiological relevance of these findings, Pep19‐2.5 was delivered via nasal aerosol to HDM‐sensitized animals during the elicitation of allergic airway inflammation and the subsequent development of experimental asthma (Figure 6G). In line with the outcome of the in vitro experiments, Pep19‐2.5 significantly reduced this inflammatory response, as evidenced by significantly improved lung function (Figure 6H) and markedly lowered numbers of inflammatory cells in both bronchoalveolar lavage (BAL) fluid and airway tissue (Figure 6I,J and Figure S7A). This was further associated with decreased levels of pro‐inflammatory cytokines (IL‐1β, TNF, IL‐6, KC), whereas IL‐4 remained unchanged (Figure 6K–O). Caspase‐1 gene and protein expression in BAL cells were slightly, but not significantly, reduced (Figure 6P,Q). Gene expression of IL‐1β was reduced by Pep19‐2.5, whereas expression of IL‐1α was not affected (Figure S7B,C). Of note, no signs of adverse Pep19‐2.5 effects (e.g., weight loss (Figure 6R) were observed during the study in any of the animals, indicating that the peptide did not induce cytotoxic effects during repetitive application over the course of the treatment. This observation is consistent with previous in vivo studies with Pep19‐2.5, including the cecal ligation and puncture sepsis model [54] and a skin wound‐healing model with six days of treatment [31], both of which reported no apparent adverse effects.

## 3 Discussion

Our study reveals how the synthetic LPS‐neutralizing peptide Pep19‐2.5 engages host‐cell membranes to selectively disrupt early events in NLRP3 inflammasome activation. These findings align with recent advances in understanding post‐translational and lipid‐dependent regulation of NLRP3, which may facilitate the development of new targeted therapeutic strategies [55]. We demonstrate that Pep19‐2.5 specifically inhibits IL‐1β secretion induced by canonical NLRP3 activators such as nigericin and MSU crystals but not inflammasomes triggered by NLRP1 or AIM2 agonists, indicating pathway specificity. Anti‐inflammatory peptides have also demonstrated preclinical efficacy in inhibiting downstream NLRP3 effectors such as GSDMD [56]. While broadly blocking GSDMD‐mediated pyroptosis may offer wide‐ranging therapeutic benefits in inflammatory diseases [57], strategies that specifically target NLRP3 potentially produce fewer off‐target effects. Mechanistically, Pep19‐2.5 interacted with host cell membranes, increasing membrane surface area, reducing membrane fluidity, and maintaining these effects over time. In contrast, the shorter peptide variant Pep19‐2.5gek showed markedly reduced interactions with model membranes and only partial inhibition of IL‐1β release, indicating that membrane engagement is a critical component of the mode of action. Given that NLRP3 activation relies on lipid‐mediated recruitment to the dTGN [9], the membrane‐modifying effects of Pep19‐2.5 are particularly relevant. NLRP3 docking to PI4P‐containing dTGN membranes is required for subsequent palmitoylation, clustering, and oligomerization [9, 10, 12]. While PI‐specific lipid binding of NLRP3 has been observed using solid‐phase lipid‐strip assays, organelle staining, and metabolic modulation of Golgi lipids [9], recombinant NLRP3 has been found to bind to phosphatidic acid (PA) and PI3P but not to all phosphoinositides, including PI4P [58]. These data support the concept that recruitment of the human NLRP3 decamer to the dTGN may not rely exclusively on PI4P association but may involve interactions with additional or alternative lipid species. Our biophysical analyses demonstrating strong Pep19‐2.5 binding to PI4P‐ and PI3P‐containing membranes, charge neutralization, and peptide accumulation in PI4P‐enriched perinuclear compartments provide a plausible mechanism by which Pep19‐2.5 could sterically or electrostatically interfere with lipid‐driven NLRP3 recruitment steps essential for inflammasome assembly. Consistent with this model, Pep19‐2.5 inhibited ASC oligomerization and speck formation, attenuated processing of pro‐IL‐1β and GSDMD, and reduced caspase‐1 activation without directly inhibiting caspase‐1 enzymatic activity. Together, these findings suggest that Pep19‐2.5 impairs an early, membrane‐proximal step in NLRP3 activation.

Pep19‐2.5 is an effective inhibitor of NLRP3‐driven inflammation in both human and murine immune cells, a pre‐requisite for pre‐clinical studies in animal models. We provide evidence for the therapeutic potential by aerosol application of the peptide in a mouse model of pulmonary allergic inflammation induced by HDM. The peptide enabled successful control of inflammation and improvement of lung function, opening new treatment options in chronic lung inflammation, which is especially important for the group of corticosteroid resistant patients with insufficient or no available treatment. Asthma triggered by inhaled allergens such as HDM is fueled by uric acid secretion in the chronically inflamed lung. Patients could benefit from the dual effects of Pep19‐2.5, the inhibitory effect of Pep19‐2.5 on HDM‐mediated activation of macrophages (Figure 6D–F) and an additional inhibitory effect on inflammasome activation by ureic acid crystals, as demonstrated in vitro (Figure 1B). Although the findings suggest mechanistic parallels between these processes, the present study did not determine the exact mechanism underlying the inhibitory effect of Pep19‐2.5 on MSU crystal‐induced IL‐1β secretion. Beyond allergic lung inflammation, membrane‐active peptides may also offer therapeutic potential for a broader spectrum of NLRP3 inflammasome‐associated diseases such as gout, metabolic inflammation, and sterile tissue injury [19, 59]. However, potential off‐target interactions with host‐cell membranes as well as other lipid‐dependent signaling pathways cannot be excluded and will require thorough evaluation in future preclinical safety studies.

In conclusion, Pep19‐2.5 may function as an “emergency brake” on NLRP3 inflammasome activation by interfering with dTGN‐associated steps required for inflammasome assembly. This previously unrecognized anti‐inflammatory mechanism of the LPS‐neutralizing peptide is distinct from that of classical small‐molecule NLRP3 inhibitors and highlights the therapeutic potential of the membrane‐active peptide Pep19‐2.5 for treating inflammasome‐driven diseases.

## 4 Experimental Section

### 4.1 Ethics statement

The studies with human blood were approved by the ethics committee of the University Clinic Bonn (315/22) and the ethics Committee of the University of Lübeck (12 202A and 2026–089). Written informed consent was obtained from all healthy donors. Animal studies were approved by the Animal Research Ethics Board of the Ministry of Environment of Schleswig‐Holstein in Kiel, Germany (V241‐38961/2017 (16‐3/23)).

### 4.2 Peptides

Pep19‐2.5 (GCKKYRRFRWKFKGKFWFWG) was purchased from Bachem (Bubendorf, Switzerland) and was produced under GMP conditions. Pep19‐2.5gek (GCKKYRRFRWKFKGK), Pep19‐2.5KO (KFGKWRFGKYRFCWKFRGWK), and N‐terminal Rhodamine‐B conjugated Rh‐Pep19‐2.5 and N‐terminal Atto488 conjugated Atto488‐Pep19‐2.5 were synthesized as described previously [26, 33, 59, 60]. NLRP3 (129–148) was synthesized by a combined semi‐automated solid‐phase peptide synthesis (SPPS) approach using the 9‐fluorenylmethoxycarbonyl (Fmoc) strategy, applying HBTU and HOBt as coupling reagents, as described before [61]. In brief, pre‐coupled H‐Phe‐2‐Cl‐Trt‐resin (1.00 mmol/g) was utilized as starting material to manually couple the respective Fmoc‐amino acids in positions 2 using 0.5 equivalents of the Fmoc‐Arg(Pbf)‐OH derivative for 60 min. Subsequent capping was performed using acetic anhydride, N‐methylimidazole, dimethyl formamide [1:2:3]. Thereby loading of the resin was reduced to 0.29 mmol/g to avoid potential peptide aggregation during synthesis. The following amino acid derivative Fmoc‐Ser(tBu)‐OH was coupled manually as described before [61]. The following amino acids of the 20mer‐peptide were coupled by automated SPPS (EPS 221 peptide synthesizer, Intavis Bioanalytical Instruments, Tübingen, Germany) using standard protocols [61]. Peptide elimination from the resin and cleavage of the side‐chain protecting groups were performed with reagent K and 95% TFA for 6 h, as reported [61]. The crude product was purified by semi‐preparative reversed‐phase HPLC to obtain the fully de‐protected peptide in high yield and purity (>95%, system: LC‐8A Shimadzu, column: Knauer Eurospher 100, mobile phase gradient system: 0.1% TFA in ddH_2_O (eluent A), 0.1% TFA in 90% MeCN (eluent B), detection: 220 nm). The peptide was analytically characterized by MALDI‐TOF‐MS (M(mono.)theor = 2695.50 g/mol, M(observed)  =  2696.67 detected as [M+H]+ on an ultrafleXtreme, Bruker Daltonics GmbH, Billerica, MA, USA) and analytical reversed‐phase HPLC (retention time: 17.3 min within a gradient of 10%–40% eluent B in eluent A in 30 min, system: LC‐10AT Shimadzu, column: Vydac 218TP,4.6 × 25 mm, 5 µm particle size, 300 Å pore size, mobile phase gradient system: 0.1% TFA in ddH_2_O (eluent A), 0.1% TFA in MeCN (eluent B), detection: 220 nm). Purity was at least 95% for all peptides.

### 4.3 Reagents

Egg chicken L‐α‐phosphatidylcholine (PC), 1,2‐dioleoyl‐*sn*‐glycero‐3‐phosphocholine (DOPC), bovine liver L‐α‐phosphatidylethanolamine (PE), porcine brain L‐α‐phosphatidylserine (PS), porcine brain sphingomyelin (SM), ovine wool cholesterol (Chol), 1,2‐dioleoyl‐*sn*‐glycero‐3‐phospho‐(1'‐myo‐inositol‐3'‐phosphate) (PI3P), 1,2‐dioleoyl‐*sn*‐glycero‐3‐phospho‐(1'‐myo‐inositol‐4'‐phosphate) (PI4P), and lissamine rhodamine B 1,2‐dihexadecanoyl‐*sn*‐glycero‐3‐phosphoethanolamine (Rh‐DHPE), were all purchased from Avanti Research (Merck, Darmstadt, Germany). N‐(7‐nitrobenz‐2‐Oxa‐1,3‐diazol‐4‐yl)‐1,2‐dihexadecanoyl‐*sn*‐glycero‐3‐phosphoethanolamine (NBD‐PE) and 1,6‐diphenyl‐1,3,5‐hexatriene (DPH) were bought from Molecular Probes (Thermo Fisher Scientific, Dreieich, Germany) and Fluka (Seelze, Germany), respectively.

### 4.4 Cell Culture

THP‐1 cells (ACC 16, DSMZ‐German Collection of Microorganisms and Cell Cultures GmbH, Braunschweig, Germany) were cultured in growth medium, consisting of RPMI 1640 (11530586, Thermo Fisher Scientific, Darmstadt, Germany) supplemented with 10% heat‐inactivated fetal bovine serum (FBS; S0615, Sigma–Aldrich, Taufkirchen, Germany), 100 U/mL penicillin and 100 µg/mL streptomycin (P4333, Sigma–Aldrich, Taufkirchen, Germany) at a density of 2–8 × 10^5^ cells/mL. Cells were used from passage 4 to 25 and maintained at 37°C under a humidified atmosphere of 5% CO_2_. THP‐1 cells were regularly tested negative for mycoplasma contamination (11‐8100, VenorGeM Classic Mycoplasma PCR detection kit, Minerva Biolabs, Berlin, Germany). For generating THP‐1 macrophages, THP‐1 monocytes were seeded into 24‐well plates at a density of 4 × 10^5^ cells/well in growth medium including 25 ng/mL PMA (phorbol 12‐myristate 13‐acetate; tlrl‐pma, Invivogen, Toulouse, France). After 48 h, adherent cells were carefully washed with PBS (phosphate‐buffered saline; P04‐53500, PAN‐Biotech, Aidenbach, Germany) and rested in PMA‐free growth medium for 24 h.

HEK293^ASC‐BFP^ cells (Institute of Innate Immunity, Bonn, Germany) were cultured in Dulbecco's modified Eagle's medium (61965026, Thermo Fisher Scientific, Darmstadt, Germany) containing 10% FBS, 100 U/mL penicillin and 100 µg/mL streptomycin. These cells were likewise kept at 37°C and 5% CO_2_.

PBMCs (peripheral blood mononuclear cells) were isolated from buffy‐coat donations (Institute of Experimental Haematology and Transfusion Medicine, University Clinic Bonn) by density gradient centrifugation using Biocoll separation media (BS L6115, Bio&Sell, Nuremberg, Germany). PBMCs were seeded into 24‐well plates at a density of 5 × 10^6^ cells/well in growth medium, consisting of RPMI 1640 (11530586, Thermo Fisher Scientific, Darmstadt, Germany) supplemented with 10% heat‐inactivated FBS (S0615, Sigma–Aldrich, Taufkirchen, Germany), 100 U/mL penicillin and 100 µg/mL streptomycin (P4333, Sigma–Aldrich, Taufkirchen, Germany), and incubated at 37°C under a humidified atmosphere of 5% CO_2_ for 1 h. Monocytes were enriched by plastic adherence. PBMCs were washed three times with PBS and non‐adherent cells were discarded.

Human macrophages were differentiated from PBMCs from the blood of healthy donors according to previously published procedures [26]. For stimulation experiments, macrophages were seeded at 10^5^ cells/well in 96‐well plates in OPTI‐MEM medium (11058021, Thermo Fisher Scientific, Dreieich, Germany) supplemented with 100 U/mL penicillin, 100 µg/mL streptomycin (P06‐07100, PAN Biotech, Aidenbach, Germany) and 2 mM L‐glutamine (59202C Sigma–Aldrich, Hamburg, Germany), and incubated at 37°C under a humidified atmosphere of 5% CO_2_ for 1 h. The human macrophages used in Figure 1B were generated as described previously [62]. Briefly, CD14^+^ monocytes were isolated from PBMCs using magnetic‐activated cell sorting (130‐050‐201; Miltenyi Biotech). The cells were seeded into 6‐well plates at a density of 10 × 10^6^ cells/well in RPMI 1640 supplemented with 10% FBS, 50 U/mL penicillin/streptomycin (all Gibco), and 50 ng/mL recombinant human macrophage CSF (M‐CSF) (11343118; ImmunoTools), and incubated at 37°C under a humidified atmosphere of 5% CO_2_ for 3 days. The resulting macrophages were seeded at a density of 0.7 × 10^6^ cells/mL in 96‐well plates.

### 4.5 Cell Stimulation

Primary monocytes, THP‐1 macrophages, and human monocyte‐derived macrophages of healthy donors were pre‐incubated with the indicated concentrations of Pep19‐2.5 for 30 min at 37°C before priming with 1 µg/mL Pam_3_CSK_4_ (tlrl‐pms‐1, Invivogen, Toulouse, France) for 3 h. Afterwards, primary monocytes and macrophages were stimulated for an additional 3 h with nigericin (tlrl‐nig) or MSU crystals (tlrl‐msu, both from Invivogen, Toulouse, France). For NLRP1 inflammasome activation assays, THP‐1 monocytes were seeded into 24‐well plates at a density of 4 × 10^5^ cells/well in growth medium containing 40 nM PMA (phorbol 12‐myristate 13‐acetate; tlrl‐pma, Invivogen, Toulouse, France). After 48 h, the adherent THP‐1 macrophages were washed with PBS (phosphate‐buffered saline; P04‐53500, PAN‐Biotech, Aidenbach, Germany) and rested in PMA‐free growth medium for 24 h. THP‐1 macrophages were incubated with the indicated concentrations of Pep19‐2.5 for 30 min, primed with 1 µg/mL Pam_3_CSK_4_ (tlrl‐pms‐1, Invivogen, Toulouse, France) for 3 h, and stimulated with 20 µM of the NLRP1 activator talabostat (tlrl‐vbp‐10, Invivogen, Toulouse, France) for 8 h. For AIM2 inflammasome activation assays, THP‐1 monocytes were seeded into 96‐well plates at a density of 1.5 × 10^5^ cells/well in growth medium containing 100 nM PMA for 3 h. THP‐1 macrophages were washed with PBS and incubated with Pep19‐2.5 for 30 min, primed with 1 µg/mL Pam_3_CSK_4_ (tlrl‐pms‐1, Invivogen, Toulouse, France) for 24 h, and stimulated with 5 µg/mL polydA:dT (tlrl‐patc, Invivogen, Toulouse, France) for 24 h.

### 4.6 Cell Viability

Effects on cell viability were assessed by MTT and LDH assays. For the MTT assay, THP‐1 macrophages were incubated with Pep19‐2.5 for 3.5 h. Subsequently, 25 µL MTT (3‐(4,5‐dimethylthiazol‐2‐yl)‐2,5‐diphenyltetrazolium bromide, 5 mg/mL) was added and incubated for 4 h at 37 °C. After removing the supernatants, DMSO (4720.1, Carl Roth, Karlsruhe, Germany) was added, and absorption at 540 nm was measured. Viability of the unstimulated cells was defined as 100%. DMSO (25% v/v; A994.1, Carl Roth, Karlsruhe, Germany) served as a positive control. Alternatively, PBMCs were isolated of blood from healthy donors, seeded at 1 × 10^6^/well in OPTI‐MEM supplemented with 100 U/mL penicillin, 100 µg/mL streptomycin, and 2 mM L‐glutamine, and incubated at 37°C under a humidified atmosphere of 5% CO_2_ for 4.5 h without or in the presence of Pep19‐2.5. After 2.5 h of incubation, MTT was added to the cells to analyze for metabolic activity. The LDH assay was performed according to the manufacturer's instructions (Thermo Fisher Scientific, Darmstadt, Germany). The percentage of LDH release was calculated compared to a 100% cell lysis control.

### 4.7 RNA Isolation, cDNA Synthesis and qRT‐PCR

Total RNA isolation was performed using innuPREP RNA mini kit 2.0 (845‐KS‐2040050, AnalytikJena, Jena, Germany) according to the manufacturer's protocol. Synthesis of cDNA was carried out using the iScript cDNA synthesis kit (1708891, Bio‐Rad, Feldkirchen, Germany). Quantitative real‐time RT‐PCR (qRT‐PCR) was performed as described [63, 64]. Primers (synthesized by Eurofins Genomics, Ebersberg, Germany) with the following sequences were used: *GAPDH* forward, 5’‐CTCTCTGCTCCTCCTGTTCGAC‐3’ and reverse 5’‐TGAGCGATGTGGCTCGGCT‐3’; *IL1B* forward 5’‐TGGAGCAACAAGTGGTGT‐3’ and reverse 5’‐TTGGGATCTACACTCTCCAGC‐3’; *TNF* forward 5’‐CCCAGGGACCTCTCTCTAATCA‐3’ and reverse 5’‐GCTACAGGCTTGTCACTCGG‐3’. Fold difference in gene expression was normalized to the housekeeping gene *GAPDH*, showing the most constant expression levels. The reaction mix containing cDNA template, primers, and SYBR green (iTaq Universal SYBR Green Supermix; 172–5125, Bio‐Rad, Feldkirchen, Germany) was run under the conditions described [64].

### 4.8 Western Blotting

Western blot analysis was performed as described previously [65]. Briefly, membranes were incubated overnight at 4°C with anti‐IL‐1β rabbit mAb (1:1000, D3U3E, #12703, Cell Signaling Technology, Leiden, The Netherlands,), anti‐cleaved‐IL‐1β rabbit mAb (1:1000, D3A3Z, #83186, Cell Signaling Technology, Leiden, The Netherlands), anti‐GSDMD rabbit mAb (1:1000, E8G3F, #97558, Cell Signaling Technology, Leiden, The Netherlands), or anti‐ASC (AL177, Biomol, Hamburg, Germany) rabbit mAb (1:1000). Anti‐rabbit IgG, HRP‐linked antibody (1:2000, #7074, Cell Signaling Technology, Leiden, The Netherlands) was incubated for 1 h at room temperature. Blots were developed with ECL reagent (Clarity Western ECL Substrate; 1705060, Bio‐Rad, Feldkirchen, Germany) and imaged using the ChemiDoc imaging system (Bio‐Rad, Feldkirchen, Germany). Values of protein expression were analyzed by densitometry and normalized to total protein levels using Image lab 6.1 (Bio‐Rad, Feldkirchen, Germany). Uncropped Western blots are shown in Figure S8.

For ASC oligomerization experiments, the cells were washed twice with HEPES (50 mM) and then lysed using a buffer consisting of HEPES (50 mM) and 0.5% Triton X‐100 (T8787, Sigma–Aldrich). After centrifugation at 6000 × *g* for 15 min at 4°C, the pellets were washed three times with HEPES (50 mM). For crosslinking, 2 mM disuccinimidyl suberate (A39267, Thermo Fisher Scientific) was added to the resuspended pellets, which were incubated at room temperature for 45 min at 37°C and centrifuged again (6000 × *g*, 15 min, 4°C). The supernatant was removed, and the cross‐linked pellets were resuspended in 100 µL of Laemmli sample buffer. Samples were boiled for 5 min at 99°C and analyzed by Western blotting.

### 4.9 Membrane FRET and Fluorescence Polarization (FP) Experiments

Liposomes of a macrophage lipid mixture PLMAK:SM:Chol 2:0.5:0.2 (M) were prepared, labeled, and analyzed at a constant temperature of 37°C on a Fluorolog SPEX (Jobin Yvon Inc., Edison, NJ, USA) as described previously [26]. Baseline signal was recorded for 50 s. Peptides were added every 50 s in increasing concentrations (FRET) or after 50 s (FP). Human macrophages of healthy donors were washed, suspended in 20 mM HEPES, 150 mM NaCl, pH 7.4, labeled with 1,6‐diphenyl‐1,3,5‐hexatriene (DPH) and analyzed immediately. The measurements were performed at 37°C in quartz glass cuvettes with a magnetic stirrer. The baseline signal in the absence of peptide was recorded for 50 s and continued after peptide addition at *t*  =  50 s. Relative fluorescence polarization was calculated as described previously [26] and indicates changes in membrane fluidity.

### 4.10 Potassium Ion Assay

Intracellular K^+^ content was determined by atomic absorption spectroscopy (AAS). THP‐1 cells were seeded into 6‐well plates at a density of 2 × 10^6^ cells/well and differentiated into macrophages as described above. After stimulation, cells were washed with potassium‐free 0.9% NaCl (H_2_O ROTIPURAN Supra, 21A2.1, Carl Roth, Karlsruhe, Germany; NaCl, 31232‐1KG‐M, Sigma–Aldrich) solution. Afterwards, cells were lysed with potassium‐free 10% HNO_3_ (HNO_3_ ROTIPURAN Supra, HN50.1, Carl Roth, Karlsruhe, Germany). Potassium content of lysates was determined by AAS (AA‐7000F, Shimadzu, Kyoto, Japan) and normalized to standards.

### 4.11 HEK293T Cell ASC Speck Formation Assay

To assess the influence of Pep19‐2.5 on ASC speck formation, HEK293T cells stably expressing ASC‐BFP fusion protein were used. MCC950 was compared to Pep19‐2.5 as a positive control. Cells were seeded into 24‐well plates at a density of 1.25 × 10^5^ cells per well. After 24 h, each well was transfected with 100 ng TetO6‐NLRP3‐hPGK‐TetON3G‐T2A‐mCherry construct using Lipofectamine 2000 (ThermoFisher Scientific) in accordance to the manufacturer's manual. This ensured moderate expression of NLRP3 and ASC speck formation. 16 h after incubation, the cells were incubated with different concentrations of Pep19‐2.5 or MCC950 and, 30 min later, NLRP3 expression was stimulated with 10 ng/mL doxycycline. After 4 h, 10 µM nigericin was added to stimulate ASC speck formation for 1 h. Cells were trypsinized, washed with DPBS and resuspended in flow buffer (DPBS, 2 mM EDTA, 0.5% BSA). ASC speck formation was measured at a LSRFortessa II and evaluated as previously described [62, 66]. The gating strategy is shown in Figure S9.

### 4.12 Specking Assay

THP‐1^C1C‐EGFP^ cells [44] (Institute of Innate Immunity, Bonn, Germany) were cultured in growth medium, consisting of RPMI 1640 (11530586, Thermo Fisher Scientific, Darmstadt, Germany) supplemented with 10% heat‐inactivated FBS (S0615, Sigma‐Aldrich, Taufkirchen, Germany) and 50 µM hygromycin (Hygromycin B, InvivoGen Europe, Toulouse, France). Cells were maintained at a density of 0.5‐2 × 10^6^ cells/mL at 37°C in a humidified atmosphere of 5% CO_2_ and used between passages 4 to 15. For microscopy experiments, THP‐1^C1C‐EGFP^ cells were seeded into poly‐L‐lysine‐coated µ‐slides (80827, Ibidi, Gräfelfing, Germany) at a density of 2 × 10^5^ cells/well and differentiated into macrophages for 48 h as described above. After differentiation, cells were washed with PBS (PAN Biotech, Aidenbach, Germany) and incubated with the indicated concentrations of Pep19‐2.5 for 30 min. Priming was performed with Pam_3_CSK_4_ for 3 h, followed by stimulation with nigericin for 1 h. The caspase‐1 inhibitor VX765 (40 µM) was added simultaneously to limit cell death. Following stimulation, cells were washed with PBS and fixed with 4% paraformaldehyde (Roti Histofix, Carl Roth, Karlsruhe, Germany). Cells were blocked with 3% BSA for 1 h and incubated overnight with an anti‐ASC rabbit mAb (AL177, 1:1000, Biomol, Hamburg, Germany). Nuclei were stained with Hoechst 34580 (H21486 Molecular Probes, Thermo Fisher Scientific) for 5 min. Prior to imaging, mounting medium (50001, Ibidi, Gräfelfing, Germany) was added. Imaging was performed using a Zeiss LSM980 with Airyscan 2 confocal microscopy. For each condition, three representative fields were selected based on the Hoechst channel. Depending on cell density, 25–69 nuclei were analyzed per field of view. C1C‐ and ASC speck formation were quantified separately using the respective fluorescence channels.

### 4.13 Fluorometric Caspase‐1 Assay

To determine the effect of Pep19‐2.5 on caspase‐1, the enzymatic activity of the enzyme in the presence or absence of the peptide was assessed. For this purpose, Pep19‐2.5 or the specific caspase‐1 inhibitor VX765 (HY‐13205, MedChemExpress, NJ, USA) was added in triplicates to the assay buffer (pH  =  7.4) consisting of 50 mM HEPES, 100 mM NaCl, 0.1% CHAPS (w/v), 1 mM EDTA, 10% glycerol (v/v), and 10 mM DTT. The fluorogenic caspase‐1 substrate Ac‐YVAD‐AMC (HY‐P2717, MedChemExpress, NJ, USA) was added to a final concentration of 10 µM. Human recombinant caspase‐1 (BML‐SE168‐5000, Enzo Life Sciences, Farmingdale, NY, USA) was added to a final concentration of 5 U/µL. Fluorescence was measured each 2 min using a Cytation5 plate reader (Biotek, Winooski, VT, USA) at 360/40 (excitation) and 450/40 (emission) over 1 h.

### 4.14 Luminometric Caspase‐1 Assay

THP‐1 monocytes were seeded into 96‐well plates at a density of 0.4 × 10^5^ cells per well and differentiated into macrophages as described above. After differentiation, cells were primed for 3 h and stimulated with nigericin for 1 h. Following stimulation, 50 µL of culture supernatant was carefully removed from each well. To maintain a 1:1 ratio of supernatant to reagent, 50 µL of Z‐WEHD reagent (Caspase‐Glo 1 Inflammasome Assay, G9951, Promega Corporation, Madison, USA) was added to each well, and plates were incubated at 37°C for 30 min. Thereafter, 75 µL of the reaction mixture, including lysed cells, was transferred to an opaque white luminometer plate. Luminescence was measured after a 30 min equilibration at room temperature using a Mithras2 LB 943. Caspase‐1‐specific activity was calculated by subtracting luminescence values obtained in the presence of the caspase‐1 inhibitor Ac‐YVAD‐CHO from those measured under uninhibited conditions. The pan‐caspase inhibitor Z‐VAD‐FMK (40 µM, tlrl‐vad, InvivoGen, Toulouse, France) was used as a control. All reagents were prepared and assays were performed according to the manufacturer's protocol.

### 4.15 NanoDSF Spectroscopy

To measure the thermal stability of NLRP3 and to detect a possible interaction with Pep19‐2.5, first nano‐differential scanning fluorimetry (nanoDSF) was performed on a Prometheus NT.48 device (NanoTemper Technologies, Munich, Germany). Thermal stability was monitored from 20°C to 90°C at a heating rate of 1.5°C/min with the excitation power set to 60%. The samples contained 3 µM NLRP3^NACHT^ and 10 µM of the respective compound (MCC950) or peptide (Pep19‐2.5). As a negative control, 3 µM NLRP3^NACHT^ without compound or peptide was measured.

### 4.16 Microfluidic Diffusional Sizing (MDS) of Pep19‐2.5 Binding

For binding experiments with recombinant human caspase‐1 (OriGene Technologies, Herford, Germany), a serial dilution of caspase‐1 (0.2–6.0 µM) was prepared and mixed with a fixed concentration (0.5 µM) of Atto488‐conjugated Pep19‐2.5. Caspase assay buffer [67] (10 mM PIPES, 10% (w/v) sucrose, 100 mM NaCl, 0.1% (w/v) CHAPS hydrate, 1 mM EDTA (pH 8.0), 10 mM DTT) was used for all dilutions. For binding experiments with recombinant human pro‐IL‐1β protein (Sino Biological, Eschborn, Germany), a serial dilution of pro‐IL‐1β ranging (0.06–4.5 µM) was prepared and mixed with a fixed concentration (2.5 µM) of rhodamine‐conjugated Pep19‐2.5. All samples were prepared on ice, incubated overnight at 4°C under gentle agitation, and measured at room temperature. Microfluidic diffusional sizing (MDS) measurements were performed on a Fluidity One‐M instrument (Fluidic Sciences, Cambridge, UK). For each condition, 4 µL of the caspase‐1/peptide or IL‐1β/peptide mixture and 4 µL of buffer as auxiliary fluid were loaded onto the microfluidic chip. Measurements were performed in at least triplicate (*n *≥ 3) using two hydrodynamic size‐range settings ((1.0–4.7) nm and (2.0–9.3) nm) and a viscosity setting of 1, corresponding to a viscosity range of (0.82–1.08) mPa s (typical of aqueous buffers). Binding affinity was quantified as the equilibrium dissociation constant *K*_D_ obtained by nonlinear least‐squares fitting using the following equation (Equation 1):

(1) $$\begin{array}{cc} R_{h} & =R_{h,\;\mathrm{free}}+\left(R_{h,\;\mathrm{complex}}-R_{h,\;\mathrm{free}}\right)\\ & \;\times \frac{\left(K_{D}+n{\left[U\right]}_{0}+{\left[L\right]}_{0}-\;\sqrt{{\left(K_{D}+n{\left[U\right]}_{0}+{\left[L\right]}_{0}\right)}^{2}-\;4n{\left[U\right]}_{0}{\left[L\right]}_{0}}\right)}{2{\left[L\right]}_{0}} \end{array}$$

Here, *R*_h_, *R*_h, free_, and *R*_h, complex_ denote the average hydrodynamic radii of the peptide–protein mixture at equilibrium, the unbound labeled peptide, and the peptide–protein complex, respectively. [U]_0_ and [L]_0_ represent the total concentrations of unlabeled protein and labeled peptide, respectively, and *n* is the number of peptide binding sites per protein molecule. The concentration of fluorescently labeled peptide was fixed at the experimental concentration, and *n* was set to 1.

For control experiments, rhodamine‐conjugated peptide at a final concentration of 2.5 µM was spiked into 10 µM of Pam_3_CSK_4_. Binding of Pep19‐2.5‐Rh was assessed by MDS on a Fluidity One‐M instrument using the same measurement settings as for pro‐IL‐1β.

### 4.17 DLS Measurements

To test for the interaction of Pep19‐2.5 with the full‐length NLRP3 protein, dynamic‐light scattering (DLS) measurements were carried out using a DynaPro NanoStar instrument Technology, Santa Barbara, CA, USA). 3 µM MBP‐tagged decameric NLRP3 (fl., wt) protein was centrifuged at 10 000 × *g* for 10 min and either measured directly or after addition of 10 µM Pep19‐2.5. The heating rate of the DLS instrument was set to 0.5°C/min, measurements were performed using single‐use cuvettes (Wyatt Technology, Santa Barbara, CA, USA) starting at 25°C, final temperature: 75°C. Per temperature, 3 single data acquisitions with acquisition times of *t*  =  5 s were recorded. The data was evaluated using the DYNAMICS software (version 7.8.3.15, Wyatt Technology, Santa Barbara, CA, USA).

### 4.18 Zeta‐Potential Measurements

Liposomes were made of DOPC, DOPC‐PI3P (95:5 M), and DOPC‐PI4P (95:5 M) at 1 mM in 20 mM HEPES, 150 mM NaCl, pH 7.5. Liposomes were suspended in Aqua ad iniectabilia (Braun Melsungen, Melsungen, Germany) to 50 µM. Zeta‐potential was determined with ZetaSizer Nano (Malvern Instruments, Herrenberg, Germany) at 25°C. The velocity (v) of 50 µM liposomes in a driving electric field with an effective voltage of 152 V was measured via dynamic light scattering and the corresponding electrophoretic mobilities (*v*/*E*) were calculated. The associated Zeta‐potentials were calculated using the Smoluchowski approximation.

### 4.19 Flow Cytometry

Membrane‐coated beads (MCBs) for flow cytometry analysis were prepared using 5 µm silica microsphere beads (Bangs Laboratories SS05003, Polysciences, Hirschberg, Germany). 1% w/v silica microspheres were washed once in 20 mM HEPES, 150 mM NaCl, pH 7.4, and incubated with 50 µL 0.5 mM DOPC liposomes (small unilamellar vesicles, SUV), containing 0, 3, 5, or 10 mol% PI3P or PI4P for 30 min in an ultrasound bath sonicator at 25°C. Afterwards, the MCBs were temperature cycled three times between 4°C and 60°C for 30 min each, washed three times to remove free liposomes and stored overnight at 4°C. To confirm the identity of PI3P and PI4P, MCBs were washed, suspended in 20 mM HEPES, 10 mM NaCl, pH 7.4, and stained with PI3P‐ and PI4P‐specific Atto488‐conjugated biosensors [46] at 200 nM for 30 min at room temperature and analyzed by flow cytometry on a Calibur flow cytometer (BD Bioscience). For peptide binding studies, MCBs were washed and suspended in 15 µL 20 mM HEPES, 10 mM NaCl, pH 7.4, and incubated with Rhodamine‐conjugated Pep19‐2.5‐Rh for 5 min at room temperature at the indicated final concentrations. After washing three times in 500 µL 20 mM HEPES, 10 mM NaCl, pH 7.4, peptide binding was quantified by flow cytometry on a Calibur flow cytometer (BD Bioscience). Data were analyzed with FCS Express Software Version 7.22.0006.

### 4.20 Confocal Microscopy Imaging

Human macrophages from healthy donors were seeded in µ‐Slides VI (80606, Ibidi, Gräfelfing, Germany) at a density of 10^5^ cells/well in OPTI‐MEM medium (11058021, Thermo Fisher Scientific, Dreieich, Germany) supplemented with 100 U/mL penicillin, 100 µg/mL streptomycin, and 2 mM L‐glutamine and incubated at 37°C under a humidified atmosphere of 5% CO_2_ for 1 h. Macrophages were primed with Pam_3_CSK_4_ for 3 h, stimulated with 10 µM nigericin for 1 h in the absence and presence of fluorophore‐conjugated Pep19‐2.5‐Rh. Active caspase‐1 was stained with the caspase‐1 pseudosubstrate FLICA660‐YVAD‐FMK (1:30, ICT9122, FLICA660 Caspase‐1 kit, Bio‐Rad, Dreieich, Germany) for 30 min at 37°C under a humidified atmosphere of 5% CO_2_. Cells were washed three times with ice‐cold caspase‐1 wash buffer, fixed for 15 min at room temperature in 4% paraformaldehyde, and washed three times in PBS. Nuclei were stained with Hoechst 34580 (H21486 Molecular Probes, Thermo Fisher Scientific, Dreieich, Germany) for 20 min at room temperature. Cells were washed three times in PBS and kept at 4°C until analysis.

Staining of membrane markers for dTGN was performed in macrophages incubated with 18 µM Pep19‐2.5‐Rh in OPTI‐MEM medium supplemented with 100 U/mL penicillin, 100 µg/mL streptomycin, and 2 mM L‐glutamine for 30 min at 37°C. Cells were washed three times with ice cold PBS (PAN Biotech, Aidenbach, Germany) and fixed in 4% paraformaldehyde in PBS for 15 min at room temperature. Staining for TGN38 [14] and PI‐lipids [46] was performed with adapted protocols. Cells were pre‐quenched twice in 100 µM glycine in PBS for 3 min and permeabilized in 10 mM PIPES buffer, pH 7.2, containing 0.1% saponin and 3% BSA for 5 min at room temperature. dTGN membranes were stained with TGN38/TGOLN2 (1:500, clone2F7.1, NB300‐575, Bio‐Techne, Wiesbaden, Germany) for 30 min at room temperature. Cells were washed three times in PIPES buffer with 0.1% saponin and 3% BSA, stained with Alexa647‐conjugated goat anti‐mouse IgG (H+L) secondary antibody (1:500, A21235, Invitrogen, Thermo Fisher Scientific, Dreieich, Germany) for 30 min at room temperature and washed three times. PIPES buffer containing 0.1% saponin and 3% BSA was used for all staining steps.

Alternatively, phosphoinositide staining was performed with PI3P‐ and PI4P‐biosensors snapped‐tagged to Atto488 at 500 nM in PIPES, 0.1% saponin, 3% BSA for 1 h at room temperature (recombinant biosensors [46] were a kind gift of Prof. Caroline Barisch). Cells were washed three times in PIPES, 0.1% saponin, 3% BSA, nuclei were stained with Hoechst 34580 for 20 min at room temperature, washed three times with PBS, and kept at 4°C until imaging. The samples were analyzed on a Leica TCS SP5 confocal laser scanning microscope (Leica Microsystems, Wetzlar, Germany) equipped with hybrid HyD detectors. Images were acquired using Leica LAS AF software (Version 2.7.3_9723).

### 4.21 ELISA

Cell culture supernatants were collected and analyzed for IL‐1β release using a commercially available ELISA kit (88‐7261‐88, Thermo Fisher Scientific, Darmstadt, Germany, and DY201, R&D Systems, Bio‐Techne, Wiesbaden, Germany).

### 4.22 Animals

Female, 8‐ to 12‐wk‐old C57BL/6 wildtype mice (Charles River Laboratories, Sulzfeld, Germany) were housed in single‐ventilated cages under specific pathogen‐free conditions. They received a diet and water ad libitum. All experiments were conducted in accordance with the German Animal Protection Law and carried out under consideration of the national guidelines for animal treatment.

### 4.23 Animal Treatment Protocols

To elicit house dust mite‐ (HDM‐) induced EAA, mice were sensitized to HDM by one intranasal (i.n.) application of 1 µg HDM extract (Greer Laboratories, Lenoir, NC, USA) in 40 µL PBS under sevoflurane anesthesia on day 1. Acute allergic airway inflammation was triggered by five consecutive i.n. challenges with 10 µg HDM extract (Greer Laboratories) in 40 µL PBS under sevoflurane anesthesia on days 8, 9, 10, 11, and 12. Pep19‐2.5 in saline (60 mg/mL) was delivered via aerosol for 20 min on days 8, 9, 10, and 11. Control mice received PBS i.n. and as aerosol. Analyses were performed 48 h later on day 14.

### 4.24 Assessment of Airway Responsiveness in Response to Methacholine

Airway reactivity was assessed by methacholine (MCh) provocation testing using invasive lung function assessment (FinePointe RC units; Data Sciences International, St. Paul, MN, USA) as previously described [68]. In brief, airway responsiveness to methacholine (MCh, acetyl‐β‐methylcholine chloride; Sigma‐Aldrich) challenge was assessed on day 14 by continuous measurement of airway resistance (RI) in anesthetized and ventilated mice. Animals were anesthetized with ketamine (90 mg/kg body weight; cp‐pharma) and xylazine (10 mg/kg BW; cp‐pharma) and tracheotomized with a cannula. Measurements were taken at baseline (PBS) and in response to inhalation of increased concentrations of aerosolized methacholine (3.125; 6.25; 12.5; 25; 50; and 100 mg/mL). After assessment of lung function, all animals were sacrificed by cervical dislocation under deep anesthesia.

### 4.25 Bronchoalveolar Lavage and Inflammatory Cell Count

BAL fluids were collected, and lungs were isolated and instilled with 1 mL ice‐cold PBS containing a protease inhibitor (Complete, Roche, Basel, Switzerland) via a tracheal cannula. The total number of immune cells was determined using a Neubauer cell counting chamber. Aliquots of BAL fluids (50 µL) were cytospinned and stained with Diff‐Quick (Medion Diagnostics, Miami, FL, USA), and leucocyte subpopulations were identified by light microscopy using morphologic criteria.

### 4.26 Lung Histology and Quantitative Morphology

Left lung lobe was dissected from mice and snap‐frozen in liquid nitrogen. Remaining lung lobes were inflated and fixed ex situ by instilling 4% (w/v) phosphate‐buffered paraformaldehyde under constant pressure for 20 min. The lungs were then stored at 4°C in paraformaldehyde overnight and subsequently embedded in paraffin. Lung orientation was randomized using the orientator technique [69]. For lung inflammation analysis, 2 µm sections were stained with hematoxylin and eosin (H&E) stain. For assessment of tissue inflammation, systematic uniform random samples of lung tissue were taken following standard methods [70]. The volume of inflammatory cell infiltrate (Vic) per total surface area of airway epithelial basal membrane (Sep) was determined using a computer‐assisted stereology toolbox (newCAST, Visiopharm, Hoersholm, Denmark) according to the following formula: Vic/Sep = LP x ∑Pic/2 x ∑Iep, where ∑Pic is the sum of all points that lie on the inflammatory cell infiltrate and ∑Iep is the sum of all intersections of test lines with the epithelial basal membrane [71]. LP is the length of the test line.

### 4.27 Assessment of Cytokines in Bronchoalveolar Lavage Fluid

Cytokine levels of murine IFN‐γ, IL‐1ß, IL‐5, IL‐6, and IL‐13 in BAL were measured using MSD U‐Plex Assays (Meso Scale Diagnostics, Rockville, MD, USA) according to the manufacturer's guidelines, with detection performed using the MESO QuickPlex SQ 120 MM (Meso Scale Diagnostics).

### 4.28 Homogenization of Lung Tissue, qRT‐PCR, and Western Blotting

Deep‐frozen lungs were homogenized with a mortar and pestle. An aliquot of 30 mg of lung powder was transferred into RLT buffer (Qiagen), and RNA was isolated with RNeasy Mini Kit (Qiagen) according to the manufacturer's guidelines. 1 µg of RNA was used for cDNA synthesis (First Strand cDNA Synthesis Kit, Thermo Fisher Scientific, Waltham, MA, USA). Quantitative RT‐PCR was performed on the Roche LightCycler 480 Instrument II system using the LightCycler 480 SYBR Green I Master (Roche Applied Science, Mannheim, Germany). Template cDNA was diluted 1:10. RT‐PCR was performed in triplicate in a total volume of 10 µL according to the manufacturer's instruction with a final primer concentration of 0.5 µM. Cycling conditions were as follows: 45 cycles at 95°C for 10 s, touch‐down annealing temperature (63°C to 58°C, temperature reduction 0.5°C per cycle) for 10 s and 72°C for 8 s. A standard curve was established by serial cDNA dilutions. The following primer sequences for reference gene *Rpl32*, *Il1a*, *Il1b*, and *Casp1* were used: *Rpl32* forward 5‘‐AAAATTAAGCGAAACTGGCG‐3’, reverse 5‘‐ATTGTGGACCAGGAACTTGC‐3’ (NM_172086.2,156 bp); *Il1a* forward 5‘‐CGCTTGAGTCGGCAAAGAAAT‐3’, reverse 5‘‐TGGCAGAACTGTAGTCTTCGT‐3’ (NM_010554.4, 98 bp); *I1lb* forward 5‘‐GAGCCCATCCTCTGTGACTC‐3’, reverse 5‘‐AGCTCATATGGGTCCGACAG‐3’ (131 bp); and *Casp1* forward 5‘‐AAACATGCGCACACAGCAAT‐3’, reverse 5‘‐CCCTCAGGATCTTGTCAGCC‐3’ (NM_009807.2, 138 bp). Negative controls (reverse transcriptase/RNA template) were included to detect possible contaminations. Amplification specificity was checked using the melting curve and agarose gel (2%) electrophoreses with a Gene Ruler 50 bp DNA ladder (Thermo Scientific). Specific mRNA levels were normalized to the level of the reference gene *Rpl32* in the same sample. Data were analyzed employing the ‘advanced relative quantification’ and ‘standard curve method’. A calibrator cDNA was included in each run to correct for run‐to‐run differences. Western blot analysis was performed on deep frozen lung tissue cells. Lung tissue was homogenized with a mortar and pestle. Lung powder was transferred into RLT buffer with ß‐mercaptoethanol, proteins were isolated, separated on SDS‐PAGE (Mini‐Protean TGX Precast Protein Gel 4–15 %; Biorad, Hercules, CA, USA), and transferred to PVDF membrane (mini 0.2 µM; Biorad). Membranes were incubated with anti‐caspase‐1 p20 antibody (AG‐20B‐0042‐C100, Biomol, Hamburg, Germany) at 1:1000 dilution or anti‐β‐actin antibody (rabbit monoclonal, A2066‐2Ml, Sigma–Aldrich) at 1:100 dilution. A 1:2000 dilution of HRP‐conjugated (Precision Protein StrepTactin‐HRP Conjugate, Biorad) goat anti‐mouse IgG1 (AB97240, Abcam, Cambridge, UK) or goat anti‐rabbit IgG1 (AB6721, Abcam) served as secondary antibodies, respectively. Immunoreactive proteins were visualized using the SuperSignal West Pico PLUS Chemiluminescent Substrate (Thermo, Waltham, MA, USA) on an iBright FL15000 Geldoc (Invitrogen, Carlsbad, CA, USA). ACBII was calculated with iBright Software (Invitrogen).

### 4.29 Statistical Analysis

All experimental data were analyzed by using GraphPad Prism 9.5.1, 10.4.2, and 11.0.0 (GraphPad Software Inc., San Diego, CA, USA). Data are presented as mean ± SEM or SD. Animal groups consisted of *n * =  10, if not stated otherwise. No statistical method was used to predetermine sample size. No data were excluded from the analyses. The experiments were not randomized. The investigators were not blinded to allocation during experiments and outcome assessment. For multiple comparisons, statistically significant differences were determined using one‐way ANOVA, followed by a Dunnett´s post‐test or Šídák's multiple comparison post hoc analysis for in vitro experiments or Tukey's post‐test for in vivo animal studies. For single comparisons significant differences were determined by an unpaired two‐sample *t*‐test. For studies of relative inhibitory effects nigericin‐ or MSU crystal‐, or HDM‐ induced IL‐1β secretion was set to 100%. All other values were calculated accordingly. Statistical differences were assessed by one‐sample *t*‐test against 100%. Curve fitting was done by four‐parameter nonlinear regression. IC_50_ values were determined for the antagonistic potency of Pep19‐2.5 on nigericin‐ or MSU crystal‐induced IL‐1β release.

## Author Contributions

J.E., N.K. A.Ke., L.P.L., D.F., H.B., I.S., C.B., A.‐K.D., A.Kl., L.B., and A.B.S performed experiments and analyzed the data. R.C.C., S.K., M.G., M.W., A.B.S., and G.W. coordinated, designed, and supervised research. J.E. drafted the initial version of the manuscript; A.B.S. and G.W. wrote the manuscript. All authors discussed and commented on the manuscript.

## Funding

The work of N.K., I.S., and G.W. is supported by the Deutsche Forschungsgemeinschaft (DFG, German Research Foundation) – GRK2873 (494832089). A.B.S. acknowledges funding by the Leibniz Research Alliance ‘Leibniz Health Technologies’ (FFS 55). A.K. is supported in parts by ‘Leibniz Health Technologies’ (FFS 55). M.G. is supported by the European Research Council (ERC Advanced Grant NalpACT) and by the DFG under Germany's Excellence Strategy–EXC2151‐390873048 and grant GE 976/16‐1. D.F. is supported by a fellowship from the Studienstiftung des deutschen Volkes, Bonn, Germany.

## Conflicts of Interest

L.P.L., M.W., A.B.S., and G.W. are inventors on a patent application (EP26188189) related to a peptide inhibitor of the NLRP3 inflammasome described in this article. R.C.C. and M.G. are advisors to BioAge Labs, and M.G. is also a co‐founder of IFM Therapeutics. The other authors declare that they have no competing interests.

## Supporting information




**Supporting File**: advs76587‐sup‐0001‐SuppMat.pdf.

## Data Availability

The data that supports the findings of this study are available in the supplementary material of this article.
