# Supplementary material for: Membrane‐Active Peptide Protects Against Inflammation by Targeting NLRP3 Activation at the Trans‐Golgi Network
Source: Adv Sci (Weinh). 2026 Jul 21:e76587. Online ahead of print. doi: 10.1002/advs.76587 (PMC13386142; doi:10.1002/advs.76587)
Supplement: Supplementary file 1 — Supporting File: advs76587‐sup‐0001‐SuppMat.pdf. [file ADVS-9999-e76587-s001.pdf]

Supporting Information

Membrane-active peptide protects against inflammation by targeting NLRP3 activation at the trans-Golgi network

Jonas Engelhardt[1], Nico Kirsch[1], Aileen Kerfin[2], Lars P. Lunding[3,4], Dominic Ferber[5], Hannes Buthmann[5], Ilka Schreier[1], Carlotta Bosio[1], Ann-Kathrin Dobbelstein[1], Anna Klawonn[1], Rebecca C. Coll[6], Lena Bauernhofer[7,8,9], Sandro Keller[7,8,9], Matthias Geyer[5], Michael Wegmann[3,4], Andra B. Schromm[2,10], and Günther Weindl[1]**

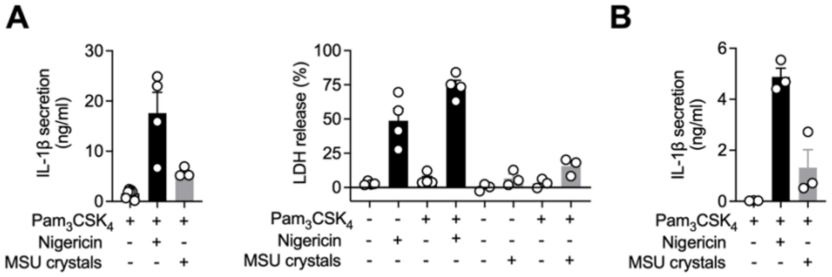

Figure S1. Stimulation of TLR2-primed cells induces IL-1β secretion and LDH release. (A) Primary monocytes or (B) THP-1 macrophages were primed with 1 µg/mL Pam_3CSK_4 for 3 h and stimulated with 10 µM nigericin or 200 µg/mL MSU crystals for 3 h. Supernatants were analyzed for IL-1β by ELISA. LDH release was determined by LDH assay. Mean + SEM (A: $n = 4$ biologically independent experiments except MSU crystals $n = 3$; B: $n = 3$ biologically independent experiments).

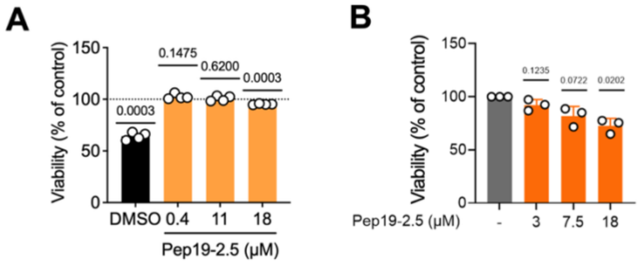

Figure S2. Cell viability of Pep19-2.5 in THP-1 macrophages and PBMCs. (A) THP-1 macrophages were incubated for 3.5 h with increasing concentrations of Pep19-2.5. Viability was analyzed using the MTT assay and normalized to non-stimulated cells (vehicle control, dashed line). DMSO (25%, v/v) was used as the cytotoxic control. Mean + SEM ($n = 4$ biologically independent experiments). One-sample *t*-test against 100%. (B) PBMCs were incubated for 4.5 h without or in the presence of Pep19-2.5. After 2.5 h of incubation, MTT was added to the cells to analyze for metabolic activity. Data are mean + SD ($n = 3$ biologically independent experiments). One-sample *t*-test against 100%.

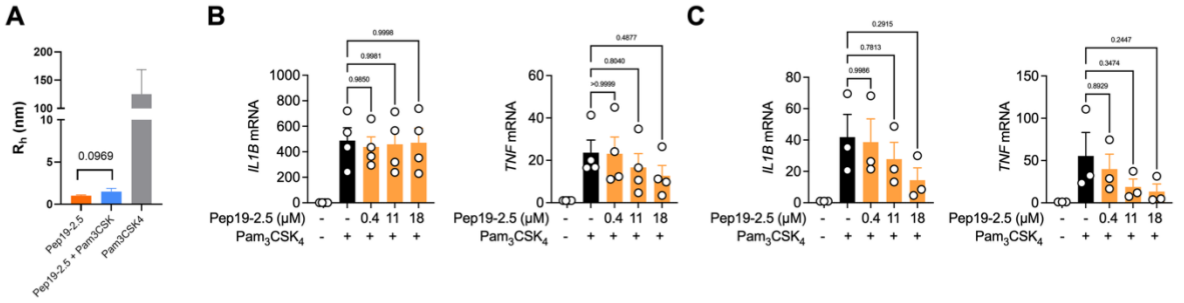

Figure S3. Pep19-2.5 does not bind to Pam_3CSK_4 and does not interfere with Pam_3CSK_4-induced cytokine expression. (A) The hydrodynamic radius (R_h) of Rh-Pep19-2.5 (2.5 µM in filtered *Aqua iniectabilia*) was determined in the absence and presence of Pam_3CSK_4 by microfluidic diffusional sizing (MDS). Data are means and SD of $n = 3$ independent experiments. Data were analyzed by unpaired *t*-test. (B) THP-1 macrophages and (C) primary monocytes were primed with 1 µg/mL Pam_3CSK_4 for 3 h. Pep19-2.5 was added 30 min before priming at increasing concentrations ranging from 0.4 to 18 µM. *IL1B* and *TNF* gene expression was normalized to housekeeping gene *GAPDH*. Bar graphs show mean + SEM ($n = 4$ biologically independent experiments for THP-1 macrophages, $n = 3$ biologically independent experiments for primary monocytes). One-way ANOVA followed by Dunnett's post-test.

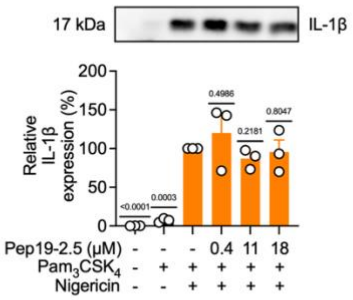

Figure S4. Pep19-2.5 does not interfere with IL-1β release. THP-1 macrophages were primed with 1 μg/mL Pam3CSK4 for 3 h and stimulated with 10 μM nigericin for 3 h. Pep19-2.5 was added 30 min before priming at increasing concentrations ranging from 0.4 to 18 μM. Protein expression of IL-1β (17 kDa) was analyzed by Western blot. Nigericin-induced expression was set to 100%. Mean + SEM ($n = 3$ biologically independent experiments). One-sample *t*-test against 100%.

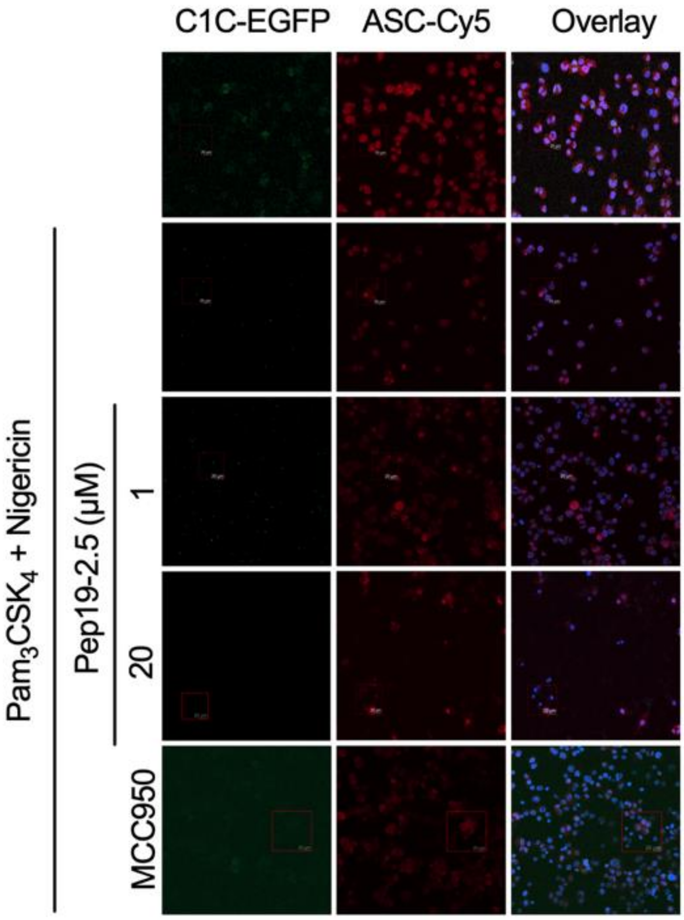

Figure S5. Pep19-2.5 inhibits NLRP3-induced ASC speck formation. THP-1[C1C-EGFP] macrophages were incubated with the indicated concentrations of Pep19-2.5 for 30 min prior to priming. After priming with 1 µg/mL Pam_3CSK_4, 40 µM VX765 was added to limit cell death, and 10 µM nigericin was added to stimulate NLRP3 assembly for 1 h. Staining was performed using an anti-ASC antibody and Hoechst 34580. Confocal microscopy images are representative of $n = 2$ biologically independent experiments, with three individual images taken for each condition. Magnified images of the indicated frames are shown in Figure 4C.

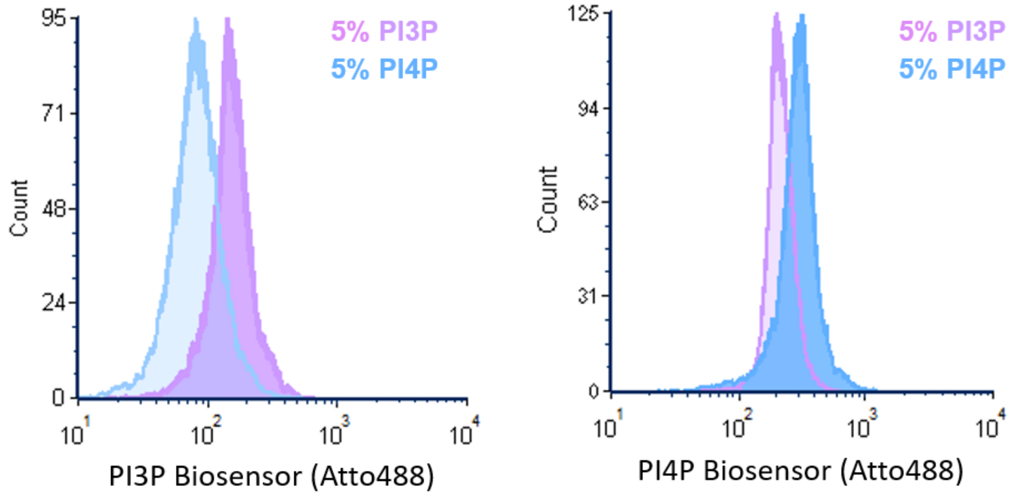

Figure S6. Staining of membrane coated beads with phosphoinositol biosensor probes. Silica beads were coated with DOPC liposomes containing 5% PI3P or 5% PI4P and stored over night at 4°C. Membrane coated beads (MCB) were stained with Atto488-conjugated biosensor probes for 30 min at room temperature and subsequently analyzed by flow cytometry. Histogramms are representative data of $n = 3$ independent bead coating and staining procedures.

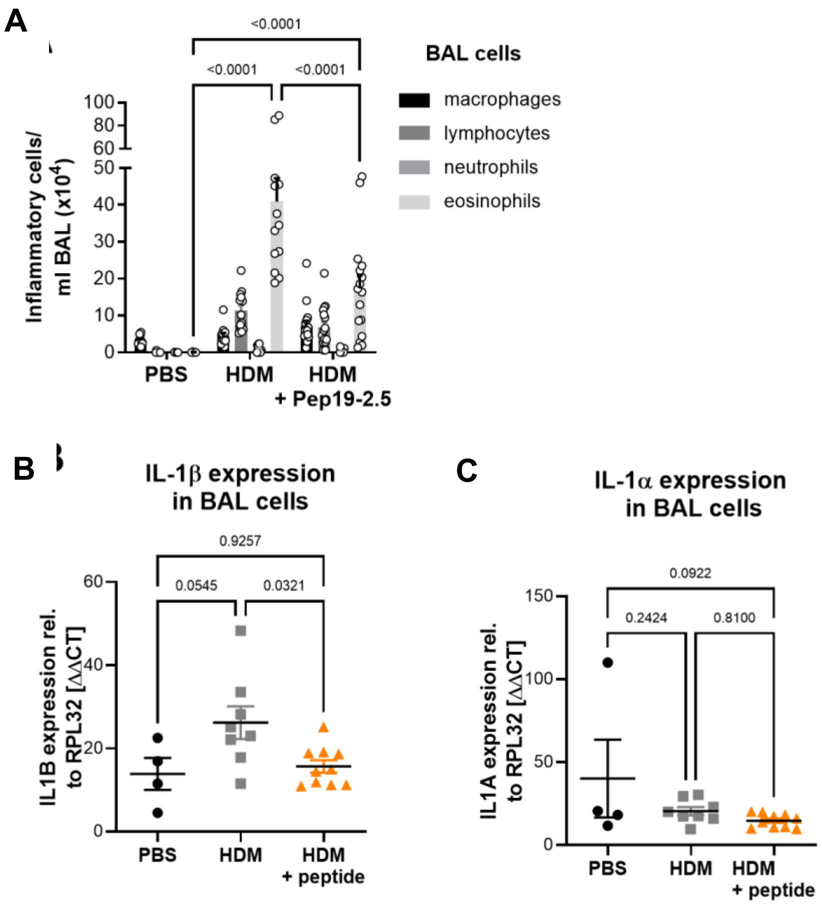

Figure S7. Pep19-2.5 reduces pro-inflammatory response to house dust mite extract *in vivo*. (A) Numbers of macrophages, lymphocytes, neutrophils, and eosinophils in bronchoalveolar lavage fluid. (B) *Il1b* and (C) *Il1a* mRNA expression in bronchoalveolar lavage cells on day 14 in healthy (PBS), asthmatic (HDM), or Pep19-2.5-treated mice (HDM + peptide), n = 10 mice per group. Results are presented as mean values ± SEM. Statistical significance was assessed using ordinary one-way ANOVA and Tukey´s multiple comparison *post hoc* analyses.

A

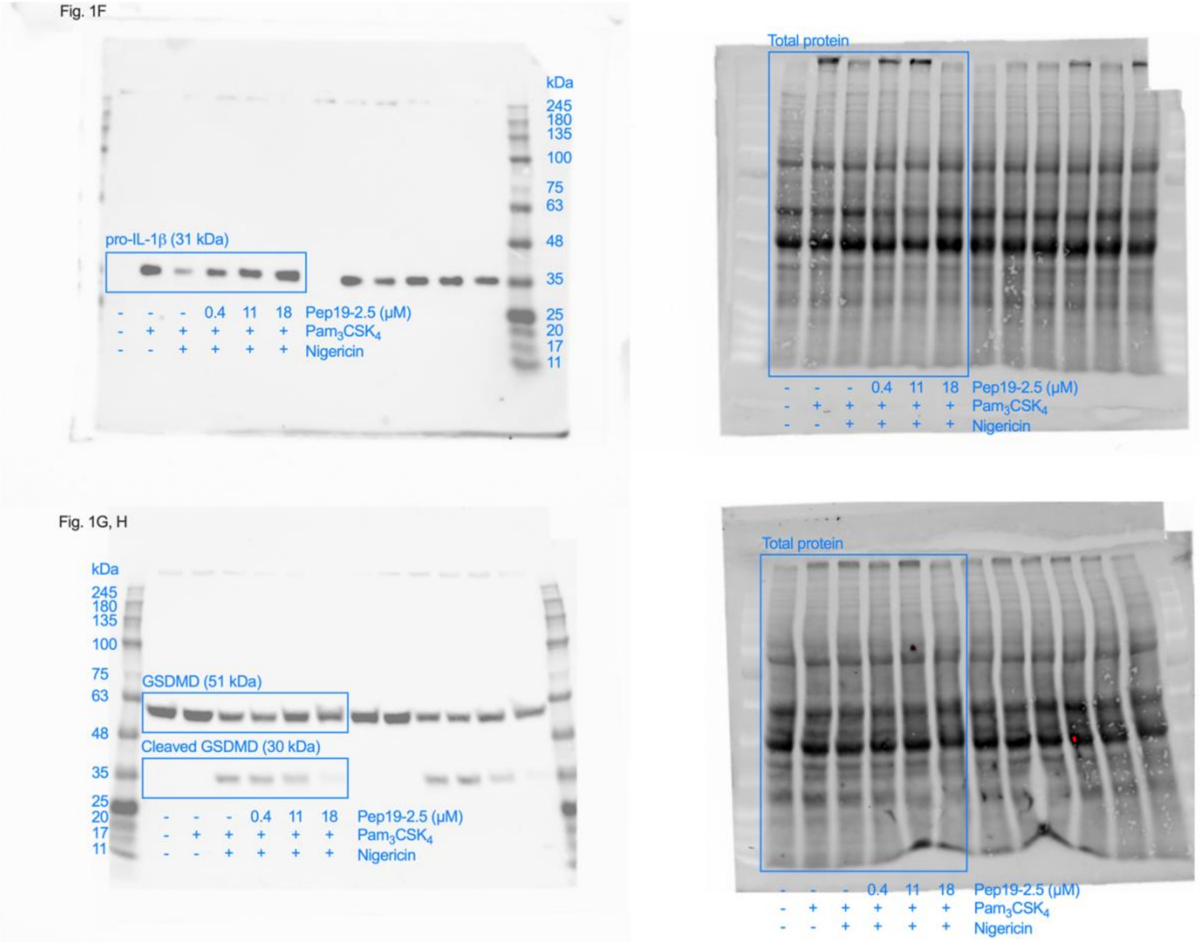
Fig. 1F
kDa
245
180
135
100
75
63
48
35
25
20
17
11
pro-IL-1β (31 kDa)
- - - 0.4 11 18 Pep19-2.5 (μM)
- + + + + + Pam$_3$CSK$_4$
- - + + + + Nigericin
Total protein
- - - 0.4 11 18 Pep19-2.5 (μM)
- + + + + + Pam$_3$CSK$_4$
- - + + + + Nigericin
Fig. 1G, H
kDa
245
180
135
100
75
63
48
35
25
20
17
11
GSDMD (51 kDa)
Cleaved GSDMD (30 kDa)
- - - 0.4 11 18 Pep19-2.5 (μM)
- + + + + + Pam$_3$CSK$_4$
- - + + + + Nigericin
Total protein
- - - 0.4 11 18 Pep19-2.5 (μM)
- + + + + + Pam$_3$CSK$_4$
- - + + + + Nigericin

B

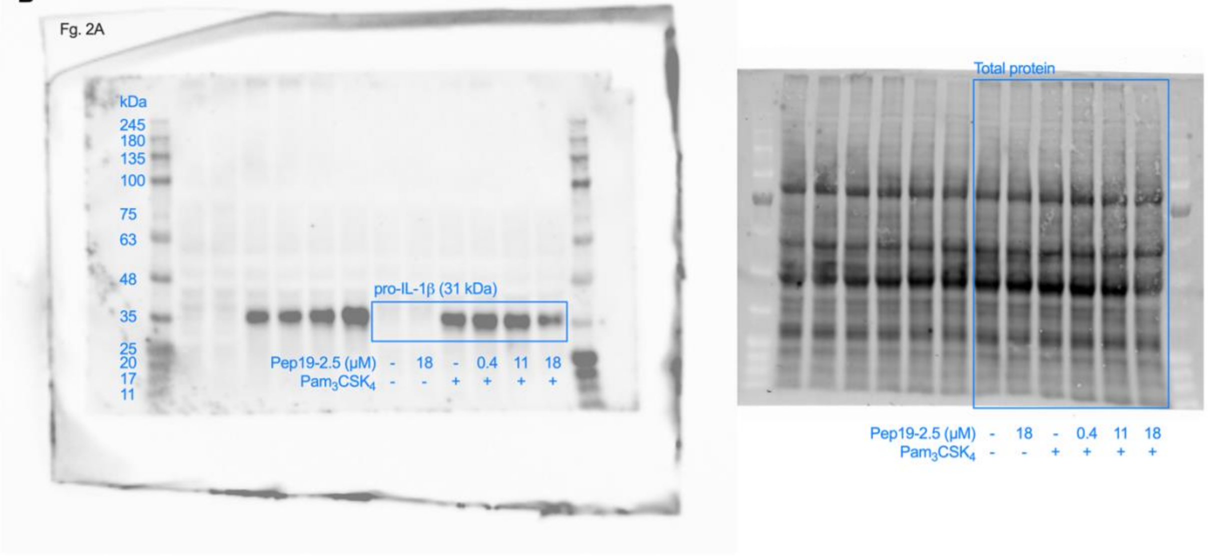
Fg. 2A
kDa
245
180
135
100
75
63
48
35
25
20
17
11
pro-IL-1β (31 kDa)
Pep19-2.5 (μM) - 18 - 0.4 11 18
Pam$_3$CSK$_4$ - - + + + +
Total protein
Pep19-2.5 (μM) - 18 - 0.4 11 18
Pam$_3$CSK$_4$ - - + + + +

C

Fig. 4A

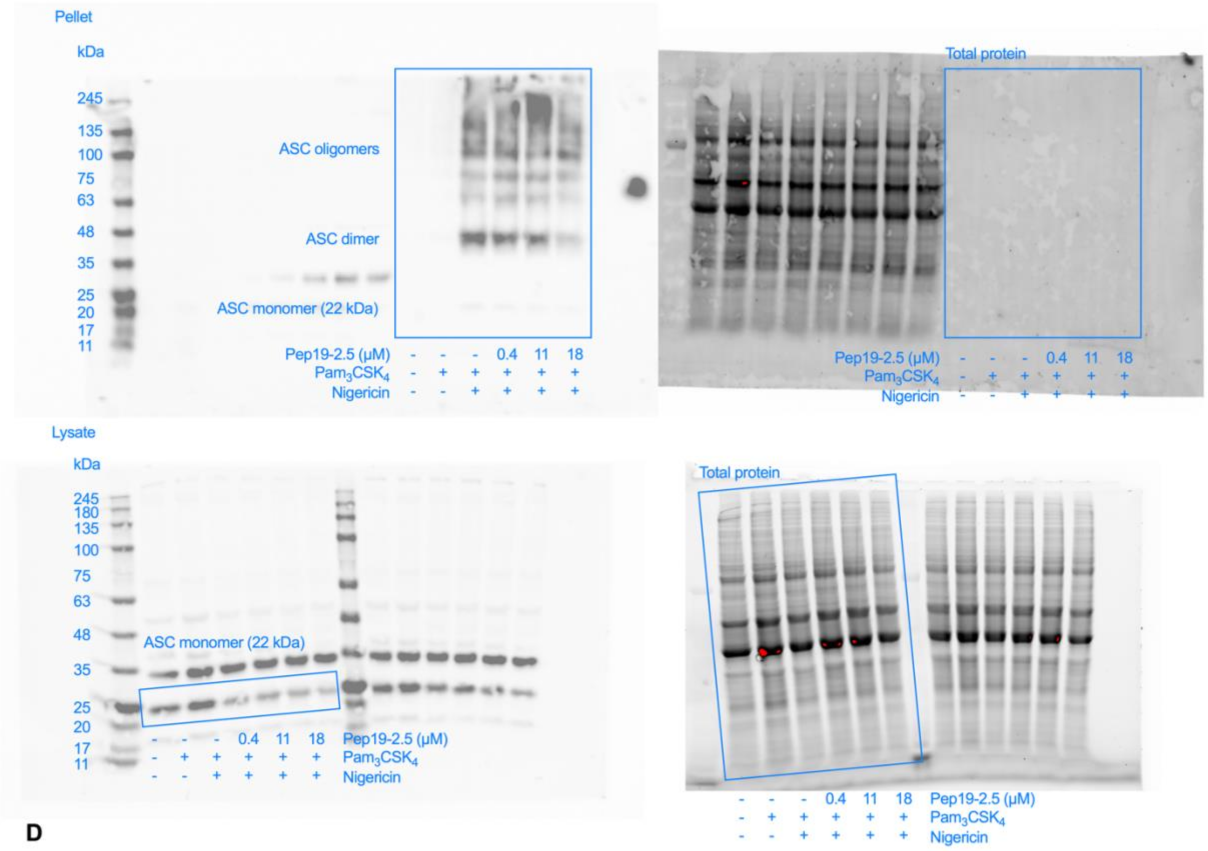

D

Fig. 6Q

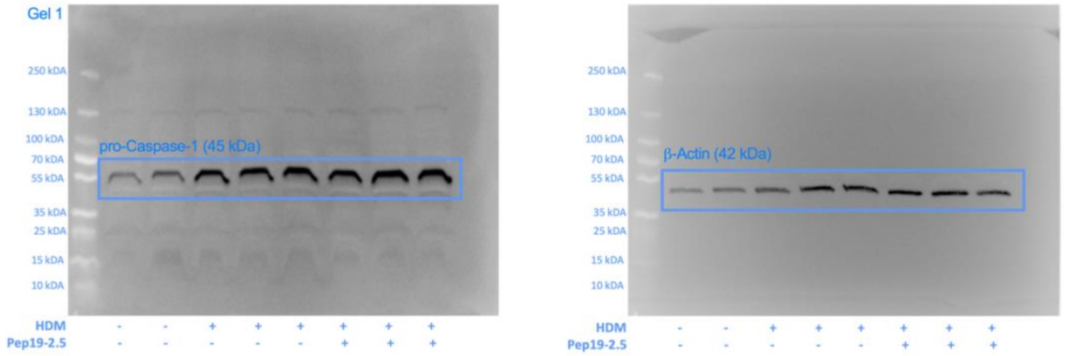

D (continued)

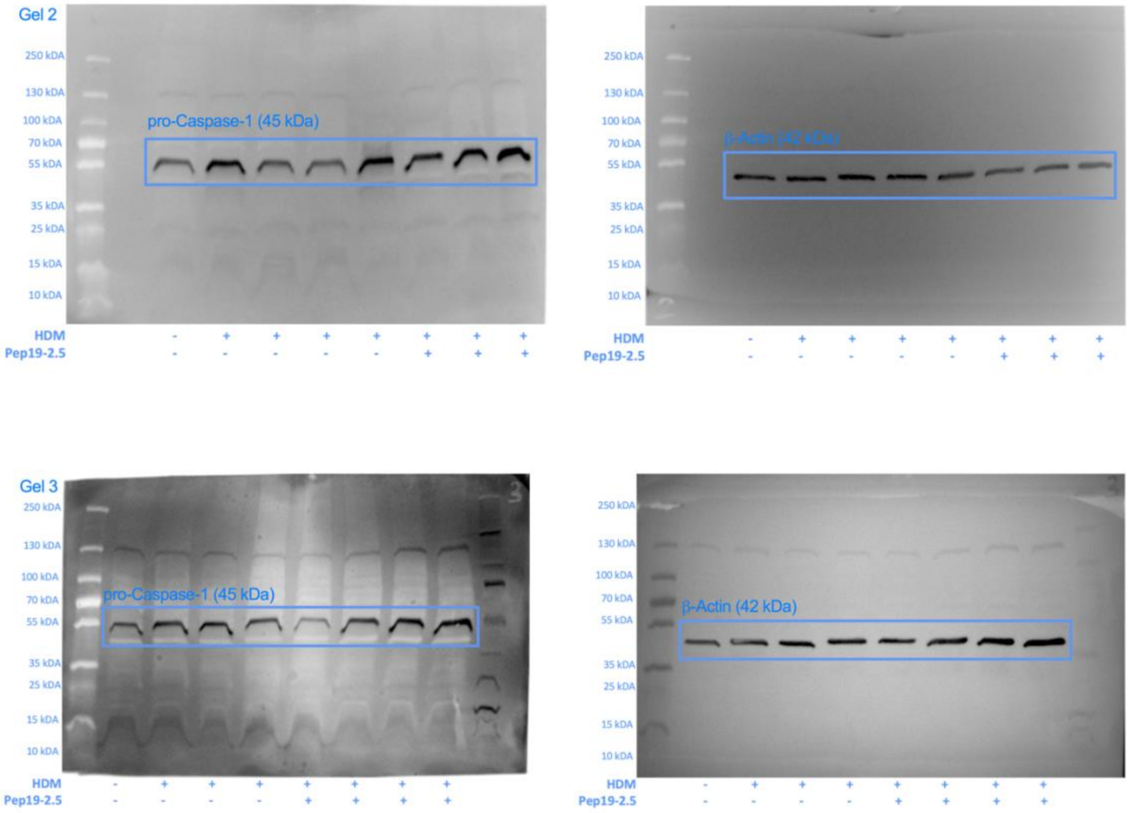

E

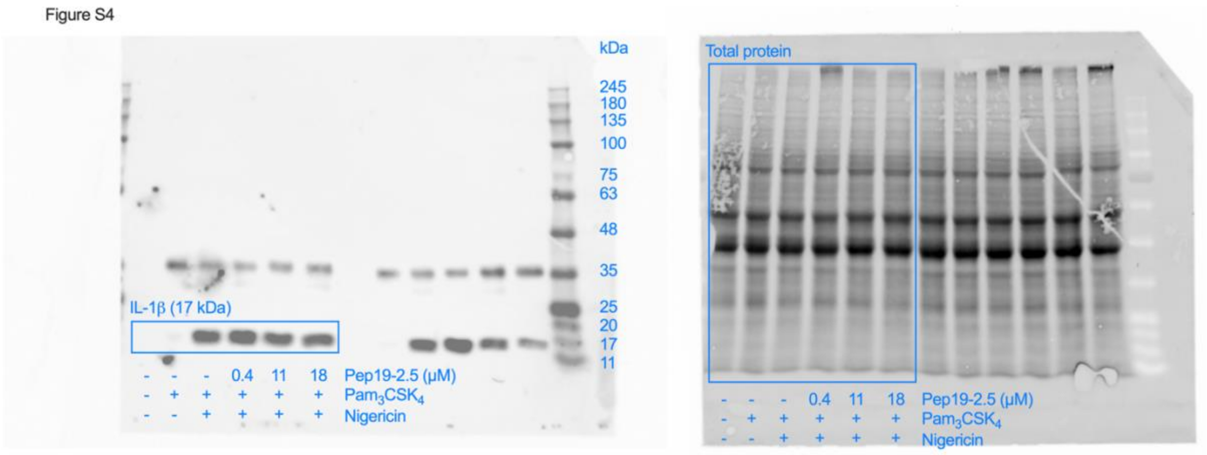

Figure S8. Whole uncropped images of original Western blots. (A) Complete Western blots of pro-IL-1β, GSDMD, and cleaved GSDMD shown in Figure 1. (B) Complete Western blot of pro-IL-1β shown in Figure 2A. (C) Complete Western blot of ASC shown in Figure 4A. No total protein bands were detected due to protein loss during ASC oligomer enrichment and crosslinking preparation, resulting in insufficient signal for fluorescence-based detection. (D) Complete Western blots of caspase-1 and β-actin, quantitative data are shown in Figure 6Q. (E) Complete Western blot of IL-1β shown in Figure S4.

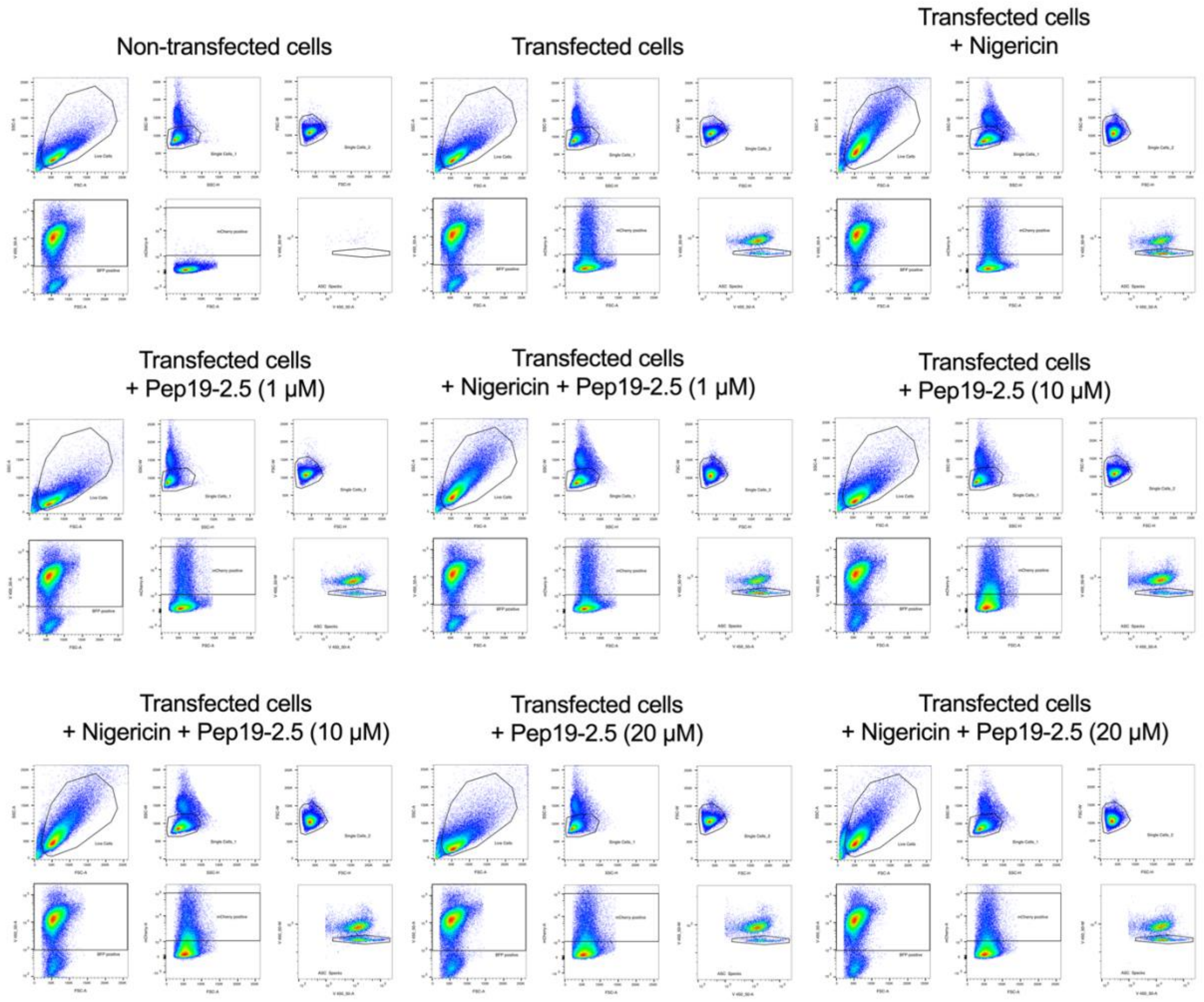

Figure S9. Gating strategy for flow cytometry analysis. HEK293$^{ASC\text{-}BFP}$ cells transduced with NLRP3 were pre-incubated with increasing concentrations of Pep19-2.5 and stimulated with or without nigericin. ASC speck formation was determined by flow cytometry, with quantitative analysis shown in Figure 4B. Dot plots are representative of n = 3 biologically independent experiments.
